# Proteomics of heterogeneous megakaryocytes identifies JI051 as an enhancer of platelet biogenesis

**DOI:** 10.26508/lsa.202603636

**Published:** 2026-09-23

**Authors:** Ryosuke Taga, Nozomi Kuwano, Yasuo Harada, Takashi Hayashi, Yuki Sakamoto, Kosuke Fujio, Yusuke Kakumoto, Emiri Nakamura, Koji Eto, Hideki Hayashi

**Affiliations:** 1 Osaka Research Center for Drug Discovery, Otsuka Pharmaceutical Co., Ltd. , Osaka, Japan; 2 Center for iPS Cell Research and Application (CiRA), Kyoto University , Kyoto, Japan; 3 Tokushima Research Center for Drug Discovery, Otsuka Pharmaceutical Co., Ltd. , Tokushima, Japan; 4 Tokushima Mima Factory, Otsuka Pharmaceutical Co., Ltd. , Tokushima, Japan

## Abstract

Induced pluripotent stem cell-derived immortalized megakaryocyte cell lines (imMKCLs) exhibit substantial heterogeneity, which poses a major barrier to efficient ex vivo production of platelet-like particles (PLPs). In this study, we combined proteomic profiling with principal component analysis to uncover regulatory factors influencing imMKCL maturation. Through correlation-based omics screening, prohibitin 2 (PHB2) was identified as a putative inhibitory node, and JI051, a small molecule previously reported to bind PHB2, was found to enhance PLP production. JI051 accelerated proplatelet-like extensions and increased PLP yield without affecting basic functional markers. Transcriptomic analysis showed that JI051 induced transcriptional changes consistent with megakaryocyte maturation and PLP biogenesis. Together, these findings identify JI051 as an enhancer of platelet biogenesis and provide a strategy to mitigate cellular heterogeneity in imMKCL megakaryocytes for scalable artificial platelet production, with potential benefits for emergency blood supply and cost-effective bioproduction.

## Abstract (precis)

This study establishes a proteomics platform to elucidate megakaryocyte heterogeneity and identifies JI051 as a compound that enhances platelet-like particle generation with bioproduction potential.

## Graphical Abstract

**Figure:**
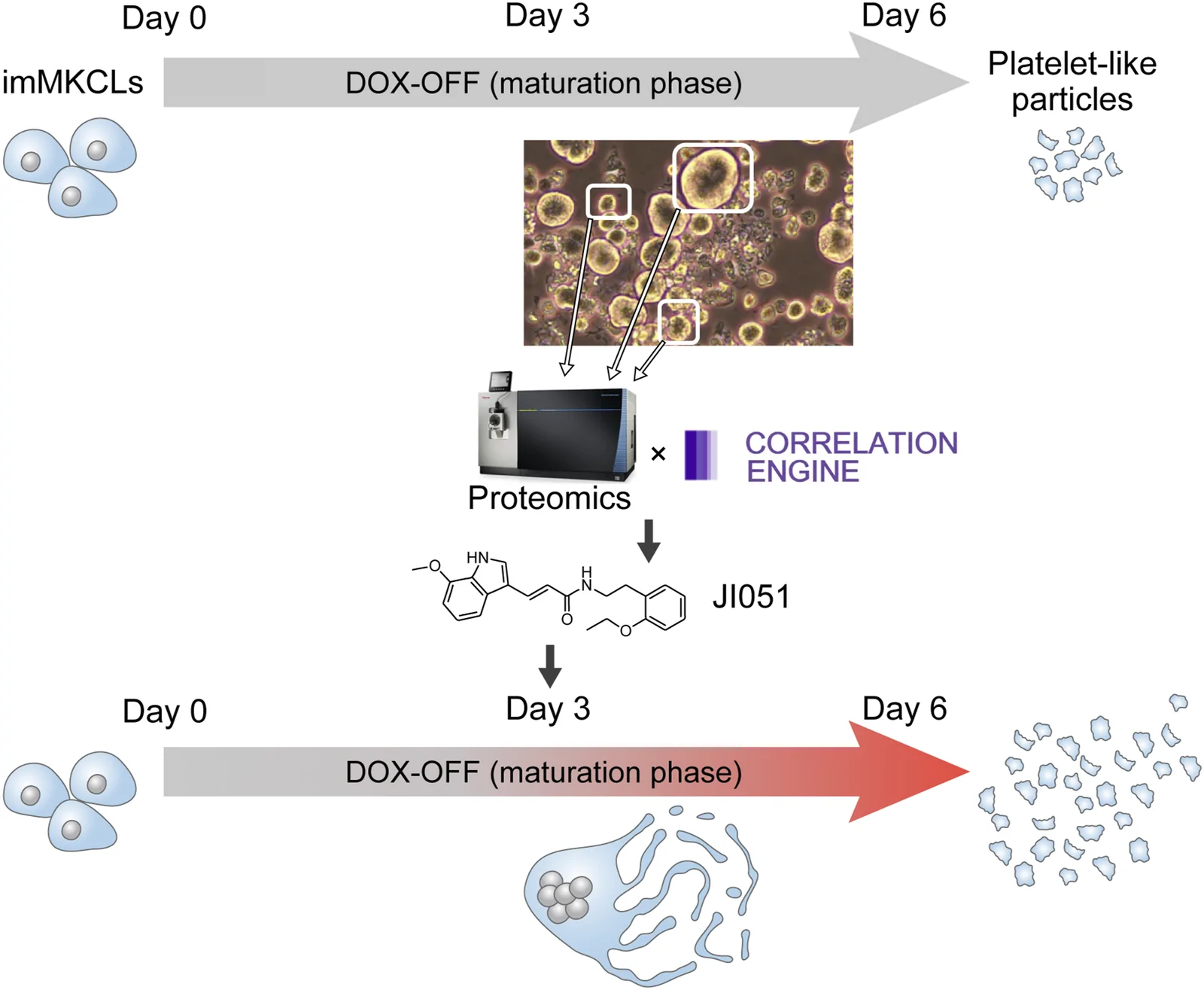

## Introduction

The COVID-19 pandemic imposed unprecedented challenges on healthcare systems and their supporting infrastructure. Conventional healthcare supply chains were disrupted, affecting the manufacture of blood-derived therapeutic products that depend on voluntary public donations (Stanworth et al, 2020; Stubbs et al, 2022). These circumstances underscored the need to establish a robust infrastructure for blood product supply that is independent of voluntary donations, even under non-emergency conditions.

Among blood products, platelet concentrates are especially vulnerable during emergencies because they cannot be cryopreserved. As such, ex vivo production of platelet-like particles (PLPs) has been a longstanding focus of research (Thon et al, 2014; Moreau et al, 2016; Sim et al, 2016; Eicke et al, 2018). However, the requirement of ∼10^11^ platelets per transfusion presents a formidable challenge for large-scale production. To address this issue, we have developed a method to generate proliferative megakaryocyte cell lines from induced pluripotent stem cells (iPSCs) and cultured them at a scale of 8 liters, achieving PLP yields at the 10^11^ level (Nakamura et al, 2014; Ito et al, 2018), thereby paving the way for artificial platelet production.

Platelets are anucleate and thus incapable of proliferation, necessitating their derivation from the cytoplasm of precursor megakaryocytes cultured at scale. Although considerable progress has been made in expanding megakaryocytes, particularly using iPSC-derived cell lines, establishing robust strategies to regulate the quantity and quality of PLP production remains a major challenge (Moreau et al, 2016; Borst et al, 2017; Sugimoto & Eto, 2021). One key obstacle lies in the heterogeneity of megakaryocyte profiles (Hegyi et al, 1991; Mattia et al, 2002). Megakaryocytes generally enlarge and form proplatelets from their demarcation membranes during maturation (Tavassoli, 1980; Schulze et al, 2006; Brown et al, 2018); however, not all cells achieve high ploidy or enlargement, and some fail to produce PLPs in vitro (Bornstein et al, 2001; Mattia et al, 2002; Chen et al, 2024). Inducing PLP production in these nonproductive megakaryocytes could substantially enhance overall yield. Therefore, we sought to identify culture additives capable of enhancing PLP production across a broader spectrum of megakaryocytes.

In the present study, we focused on cell size in iPSC-derived immortalized megakaryocyte cell line (imMKCL) and conducted proteomic analysis on size-stratified cell populations. We identified seven candidate proteins potentially involved in megakaryocyte maturation, among which the chemical compound JI051, targeting prohibitin 2 (PHB2), markedly enhanced PLP production.

## Results

### Subtle proteomic differences define size-based heterogeneity in imMKCL megakaryocytes

To identify culture additives that enhance PLP yield through simple supplementation during megakaryocyte cultivation, we used the imMKCL, derived from iPSCs (Takayama et al, 2010; Nakamura et al, 2014). This cell line proliferates during the “ON” phase, in which the expression of three transgenes (*c-MYC*, *BMI1*, and *BCL-XL*) is transiently induced via a tetracycline-regulated system. Upon cessation of transgene expression (“OFF” phase), the cells exit the proliferative state and initiate maturation, culminating in PLP production over ∼6 d.

**Figure 1.**
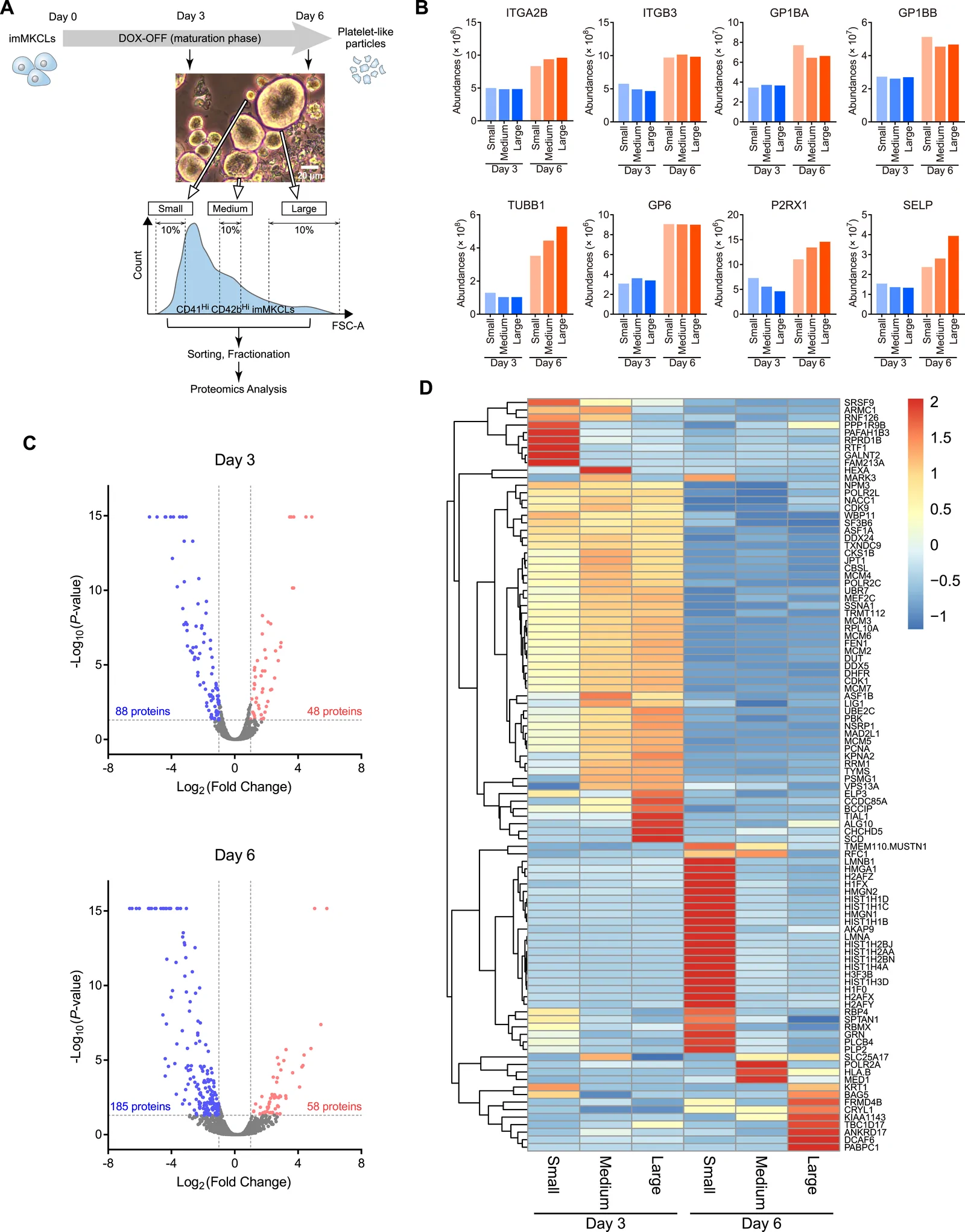
Subtle proteomic differences define size-based heterogeneity in iPSC-derived megakaryocytes. **(A)** Schematic diagram of the experimental workflow for proteomic analysis. imMKCL megakaryocytes were separated into six samples according to cell size and culture duration, after which the cytoplasmic fraction was isolated and subjected to quantitative proteomics. **(B)** Relative expression profiles of representative megakaryocyte-associated proteins identified from the proteomics dataset. **(C)** Volcano plots comparing the abundance of 3,533 quantified proteins between Small and Large cells at Day 3 (top) and Day 6 (bottom). Proteins with at least a twofold difference and *P* < 0.05 are highlighted in red (to indicate proteins up-regulated in Large cells) or blue (to indicate proteins up-regulated in Small cells). **(D)** Heat map of the 100 proteins with the highest coefficients of variation across all samples.

During maturation, cell enlargement occurs heterogeneously; some cells enlarge and produce PLPs, whereas others remain small and fail to produce PLPs (Fig 1A). To enhance PLP production efficiency, we focused on mitigating this heterogeneity. Specifically, we aimed to induce PLP production in megakaryocytes that typically do not contribute to output, thereby increasing overall yield.

We hypothesized that distinguishing high-yielding (“productive”) from low-yielding (“nonproductive”) megakaryocytes based on phenotypic characteristics could inform strategies to improve population-wide PLP output. Because PLP production is a downstream consequence of megakaryocyte maturation, retrospectively linking individual cell traits to output is inherently challenging. Therefore, we used cell enlargement, a widely accepted morphological proxy for maturation, as a surrogate marker of PLP production capacity (Hegyi et al, 1991). Indeed, within imMKCL megakaryocytes, larger cells tend to produce a greater number of PLPs (Nakamura et al, 2025).

**Figure S1.**
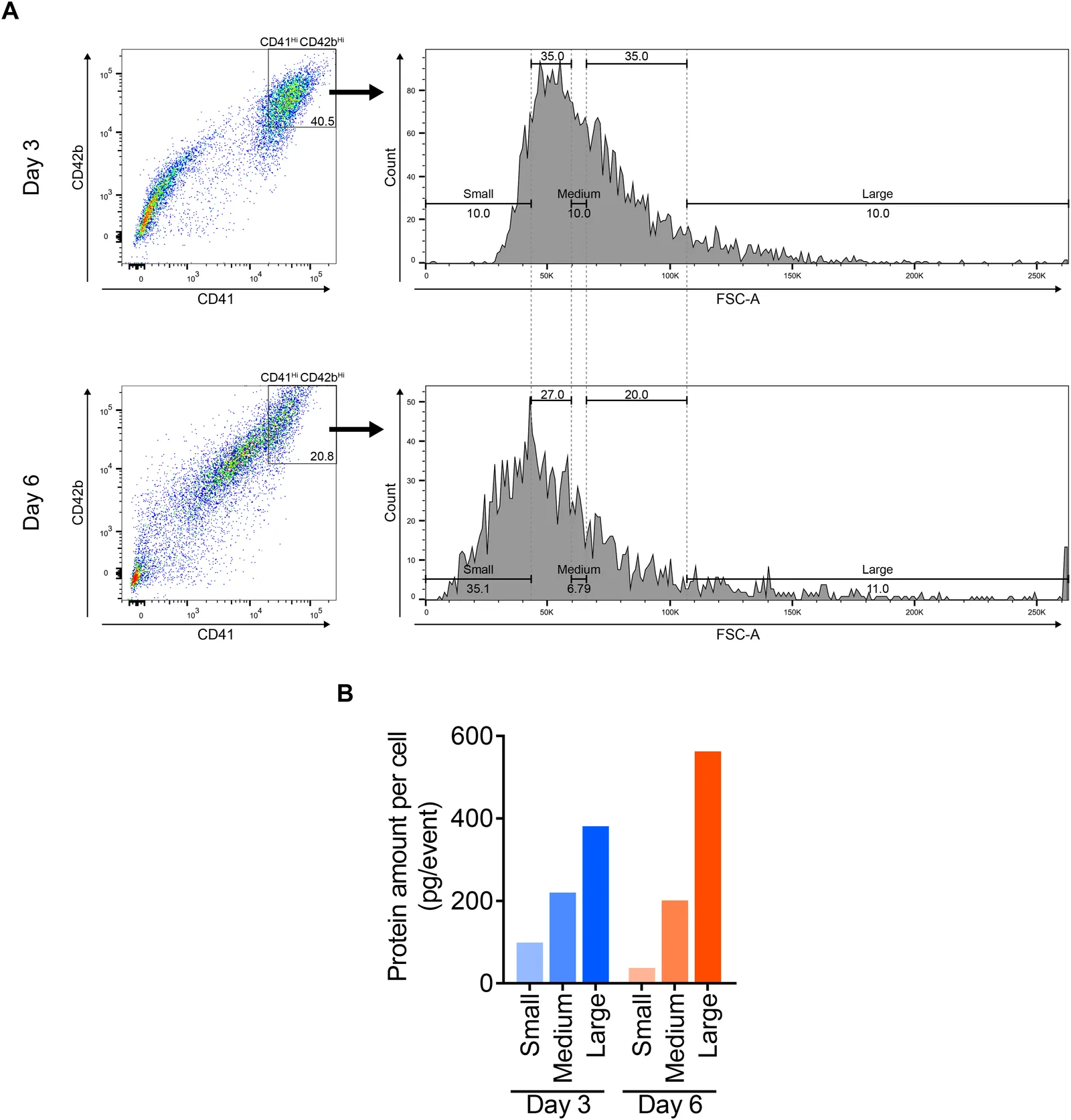
Sorting strategy of the imMKCL megakaryocytes. **(A)** Gating strategy. Gates corresponding to the top 10%, middle 10%, and bottom 10% of the FSC distribution were defined for the CD41^Hi^/CD42b^Hi^ population on Day 3, and the same gate boundaries were applied to the Day 6 samples. **(B)** Protein amount per sorted event. This value was calculated from the total protein amount extracted from each sample and the number of events recorded during sorting.

We performed FACS analysis on the imMKCL cells at Day 3 of the OFF phase, sorting the cells by forward scatter (FSC), which correlates with cell size. The top 10% of FSC values were designated as “large cells,” the middle 10% as “medium cells,” and the bottom 10% as “small cells” (Figs 1A and S1A). The same sorting procedure was applied to Day 6 cells using the FSC gate boundaries defined at Day 3, without adjustment for differences in FSC distribution. This yielded six distinct populations: Day 3–Small, Medium, Large; Day 6–Small, Medium, Large. Each population was lysed, and soluble proteins were extracted. To confirm that sorting had successfully enriched for the intended cell-size fractions, we calculated the amount of extracted protein per sorted event using the measured total protein yield and the recorded number of events. The resulting protein-per-event values increased with the FSC gates, indicating that larger FSC fractions contained cells with greater protein content and supporting the conclusion that the collected cell fractions indeed reflected the targeted cell sizes (Fig S1B).

The extracted proteins from each population were then analyzed by mass spectrometry. A total of 3,533 proteins were identified in the imMKCL proteome (Table S1), including key megakaryocyte and platelet markers such as ITGA2B, ITGB3, GP1BA, and GP1BB (Fig 1B). The 10 most abundant proteins in the most mature group (Day 6–Large) showed partial overlap (6/10 proteins) with those in the least mature group (Day 3–Small) (Table S2). Notably, HSP90β, which was highly expressed in Day 3 samples, declined in Day 6 samples, whereas ITGB3 expression increased. In addition, cytoskeletal proteins such as beta-tubulin were more abundant in Day 6 samples than in Day 3 samples (Fig 1B).

Table S1. Processed data of the proteomic analysis in each cell population of the imMKCL.

Table S2. Top 10 most abundant proteins identified by proteomic analysis in each cell population of the imMKCL.

Comparison of expression patterns between Small and Large cells at both Day 3 and Day 6 revealed that only a small fraction of proteins exhibited more than a twofold change, whereas the vast majority showed less variation, with no substantial differences in known maturation-related proteins (Fig 1B and C). Although megakaryocyte marker proteins generally increased from Day 3 to Day 6, no consistent trend was observed with respect to cell size alone (Fig 1B). Visualization of the top 100 proteins with the highest coefficient of variation revealed enrichment of DNA replication and cell cycle–related proteins in Day 3–Medium and Day 3–Large cells, whereas Day 6–Small cells were enriched in nuclear proteins such as histones (Fig 1D).

### Proteome-based analysis identifies PHB2 as a candidate inhibitory node for megakaryocyte maturation

**Figure 2.**
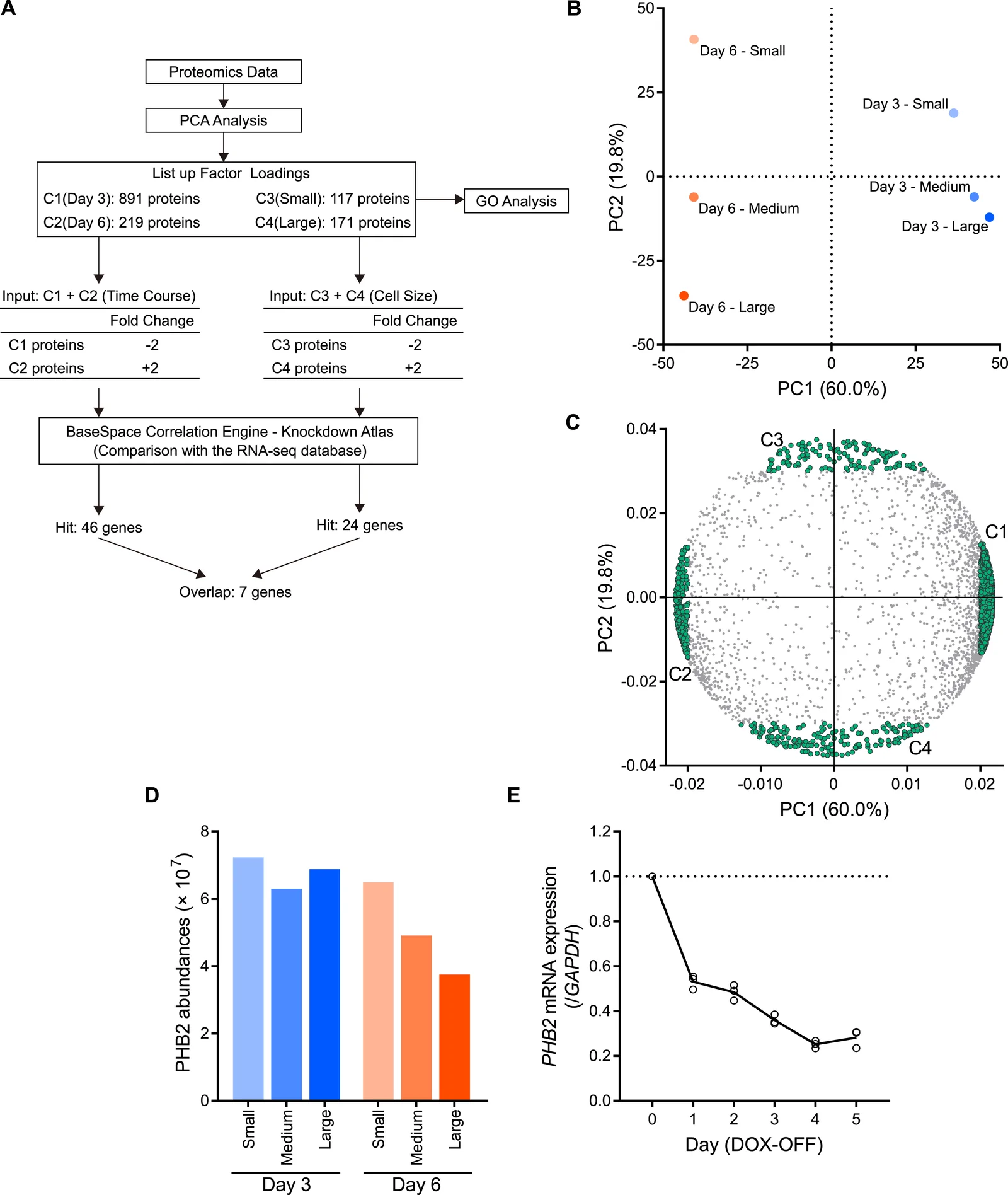
Proteome-based principal component analysis identifies PHB2 as a candidate inhibitory factor in megakaryocyte maturation. **(A)** Schematic diagram of the analysis workflow. Principal component analysis (PCA) was applied to the proteomic dataset, and four protein lists were generated based on factor loadings for PC1 and PC2. Proteins in each list were then assigned weights of +2 or −2, followed by gene ontology (GO) analysis or Knockdown Atlas analysis (Correlation Engine). Seven candidate genes were identified from the intersection of the resulting hits. **(B)** PCA plot of the six samples. **(C)** Plots of protein contributions (factor loadings) to the principal component axes. Proteins included in the lists are shown as green dots. **(D)** Expression pattern of PHB2 in the proteomics dataset. **(E)** Relative mRNA expression of *PHB2* over time during maturation of the imMKCL cells under DOX-OFF conditions.

We performed a comprehensive analysis of protein expression profiles across six sorted cell populations: Day 3–Small, Day 3–Medium, Day 3–Large; and Day 6–Small, Day 6–Medium, Day 6–Large (Fig 2A). Principal component analysis (PCA) was performed, and the first two principal components (PC1 and PC2) were examined. PC1 effectively distinguished Day 3 from Day 6 samples, suggesting that proteins contributing to this axis are primarily influenced by culture duration. PC2 differentiated Small from Large cells, indicating that proteins along this axis are associated with cell enlargement (Fig 2B).

**Figure S2.**
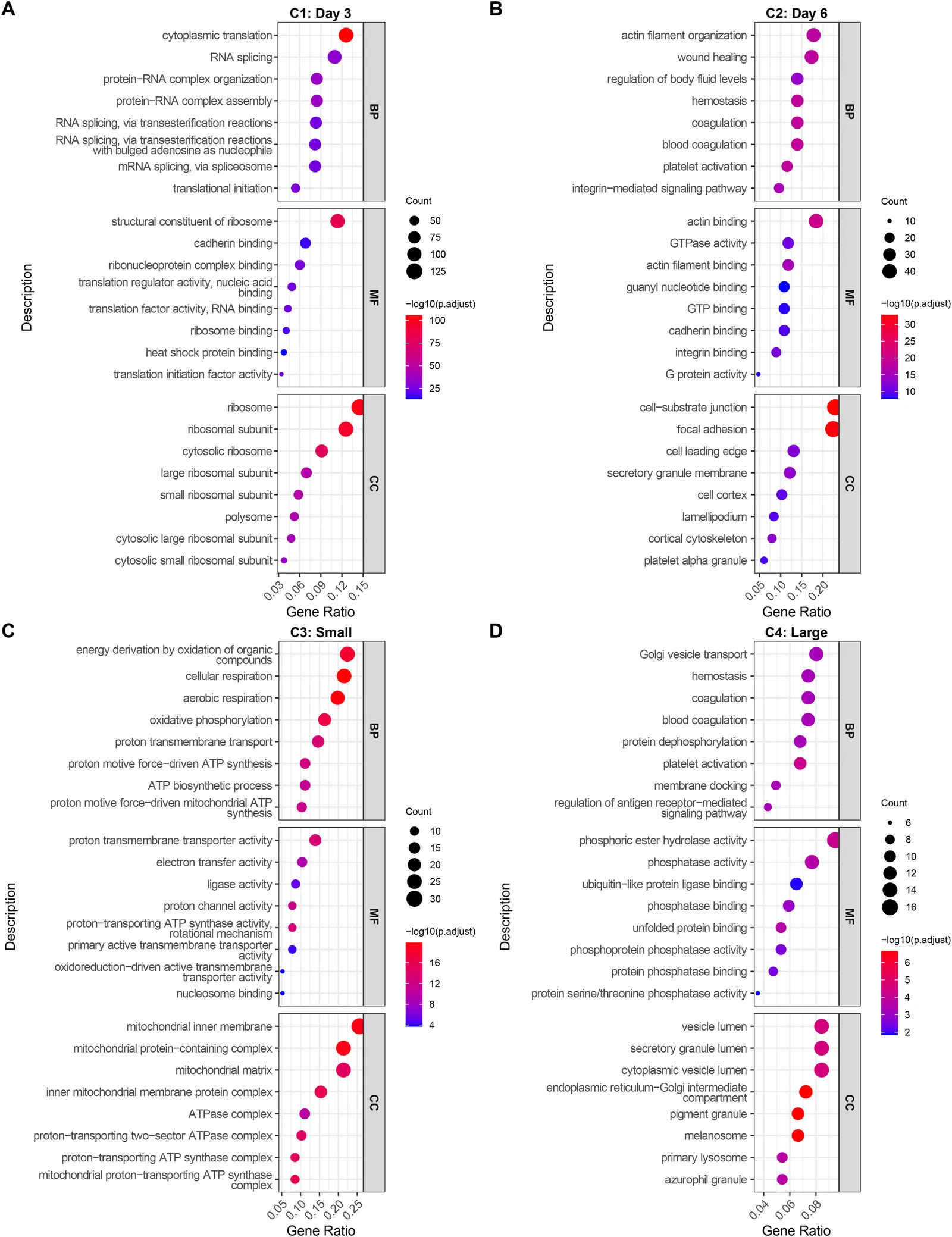
GO enrichment analysis of four protein lists derived from PCA factor loadings. **(A, B, C, D)** GO enrichment plots for each protein set. **(A)** C1: Day 3, (B) C2: Day 6, (C) C3: Small cells, and (D) C4: Large cells. BP, biological process; MF, molecular function; CC, cellular component.

Proteins with high absolute factor loadings on each axis were categorized into four groups. Each group was organized based on cell population, as follows: C1 (Day 3 samples), C2 (Day 6 samples), C3 (Small samples), and C4 (Large samples) (Fig 2C). Gene ontology (GO) enrichment analysis revealed that C1 was enriched in transcription and translation-related terms, including mRNA and ribosomes (Fig S2A); C2 included markers of platelet function, such as the actin cytoskeleton, G proteins, hemostasis, aggregation, and α-granules (Fig S2B); C3 was associated with respiration-related terms such as ATP production and mitochondrial function (Fig S2C); C4 was enriched in platelet-related terms, membrane transport, secretion, and endoplasmic reticulum–Golgi pathways (Fig S2D). These findings indicate that Day 3 cells represent the early to mid-stages of maturation, marked by polyploidization and endomitosis, whereas Day 6 cells are more committed to platelet production. The enrichment of membrane-related GO terms in C4 (Large cells) may be indicative of a state compatible with readiness for platelet release. Thus, the PCA-derived protein groups (C1–C4) effectively capture the progression of megakaryocyte maturation.

We hypothesized that megakaryocyte maturation could be facilitated by shifting protein expression from C1/C3–associated profiles toward the negative end of both PC1 (temporal axis) and PC2 (size axis). Specifically, down-regulating C1 and C3 proteins (immature markers), while up-regulating C2 and C4 proteins (mature markers) was expected to promote maturation.

Using the BaseSpace Correlation Engine platform (Illumina), we interrogated public gene expression datasets to identify knockdown targets capable of down-regulating C1/C3 proteins and up-regulating C2/C4 proteins (Fig 2A). We identified 46 candidate targets for knockdown predicted to suppress C1 and enhance C2 expression, and 24 candidate targets predicted to suppress C3 and enhance C4 expression (Tables 1, S3, and S4). The following seven genes were common to both lists: *PAN2*, *PHB2*, *ATP6V0B*, *TNFRSF21*, *GNAS*, *ARG1*, and *AKR1A1*.

**Table 1.** Candidate knockdown genes predicted to promote imMKCL maturation based on Correlation Engine Knockdown Atlas analysis (related to Tables S3 and S4).

Input: C1+C2 (Dox-OFF time course)					Input: C3+C4 (cell size)		
*ST6GALNAC2*	*CASP8AP2*	*MCOLN1*	*BLM*	*VSIG1*	*PAN2*	*NBN*	*GMEB2*
*PKIG*	*CRADD*	*MAPK13*	*CLASRP*	*AKR1A1*	*GNAS*	*EZH1*	*ARG1*
*FUBP3*	*IPO11*	*ATP6V0B*	*ARG1*	*AGL*	*AKR1A1*	*PGM1*	*SNRPC*
*RHOBTB1*	*KIF14*	*TNFRSF21*	*GRHPR*	*GAS1*	*ATP6V0B*	*CRELD2*	*DVL2*
*UBR7*	*PEPD*	*ASL*	*PPP2R3C*	*SLC11A2*	*TNFRSF21*	*SLC39A8*	​
*PAN2*	*PPP1R15B*	*BID*	*PPIC*	*PHKG2*	*UGT2B28*	*DPF1*	​
*HTRA1*	*SLC2A1*	*FAM20B*	*CTTN*	​	*PHB2*	*EPRS*	​
*DHPS*	*TCFL5*	*EPHB4*	*LYRM1*	​	*DLD*	*NAA50*	​
*PHB2*	*ROR1*	*GNAS*	*PMM2*	​	*TM9SF2*	*AXIN1*	​
*RASSF2*	*NRP2*	*MRPL19*	*DECR1*	​	*DAP*	*PTPN5*	​

Table S3. List of hit genes identified from Knockdown Atlas analysis based on the C1+C2 dataset (related to Table 1).

Table S4. List of hit genes identified from Knockdown Atlas analysis based on the C3+C4 dataset (related to Table 1).

**Figure S3.**
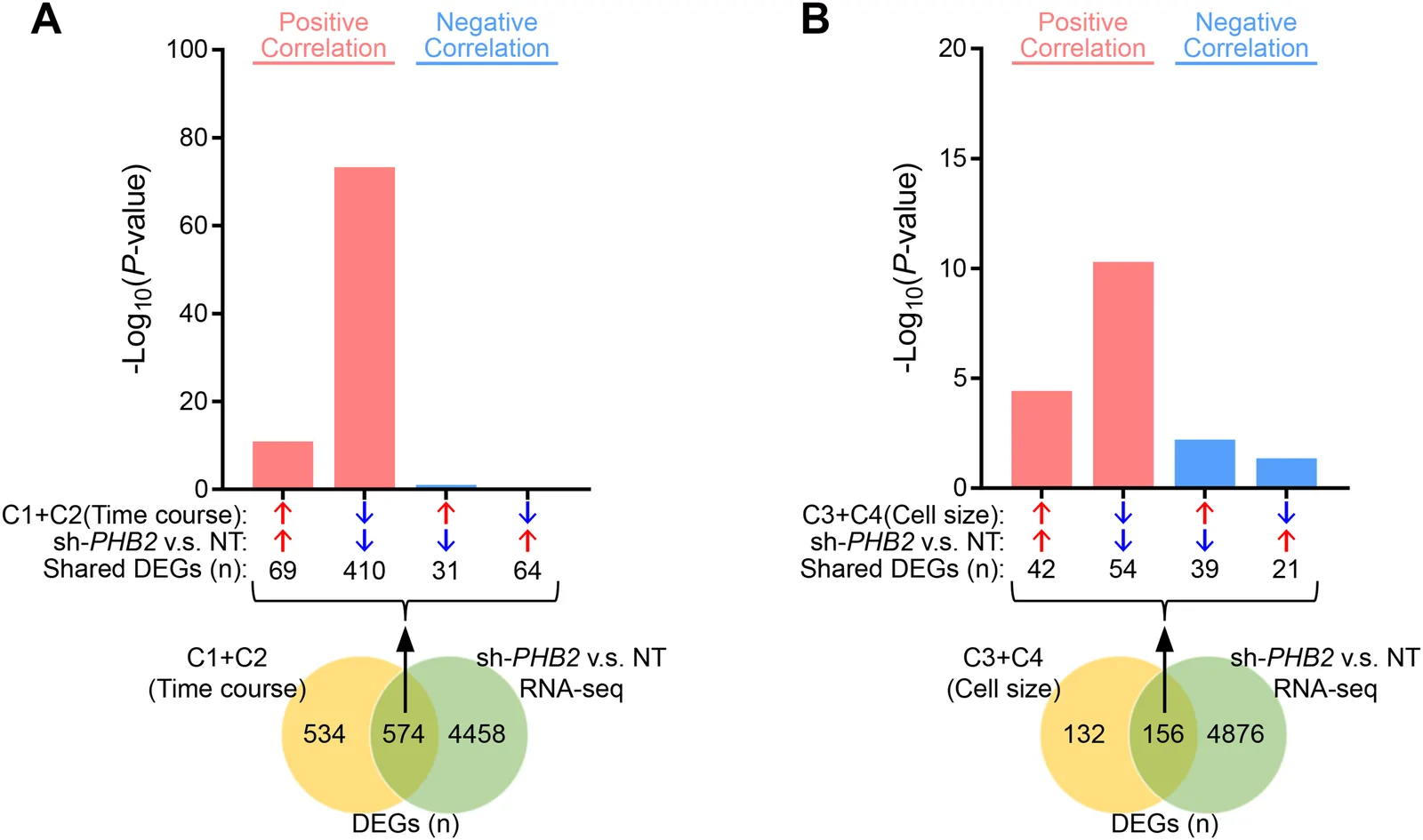
Correlation plots between *PHB2* knockdown datasets and proteomic profiles during imMKCL maturation. **(A, B)** Correlation plots comparing the *PHB2* knockdown dataset with (A) the C1+C2 dataset (culture duration) and (B) the C3+C4 dataset (cell size). DEGs, differentially expressed genes; NT, non-targeting shRNA.

Among these, we focused on *PHB2* (Fig S3A and B) because a related study performed RNA sequencing (RNA-seq) analysis of *PHB2* using human primary hematopoietic stem/progenitor cells (Liu et al, 2017). PHB2 is broadly distributed across the nucleus, cytoplasm, and mitochondria and interacts with various transcription factors, including nuclear receptors. It also contributes to the regulation of mitophagy (Bavelloni et al, 2015). The referenced study reported that PHB2 expression increases during erythroid differentiation, and its knockdown impairs erythropoiesis (Liu et al, 2017).

However, data on the role of PHB2 in megakaryocytes and platelets are limited, primarily implicating this protein in platelet activation (Zhang et al, 2012; Hu et al, 2021). Our proteomic analysis revealed that PHB2 expression declined with increasing cell size at Day 6 (Fig 2D). Quantitative RT–PCR analysis showed that *PHB2* mRNA levels decreased by 50% from Day 0 to Day 1 and further declined to ∼25% by Day 4 (Fig 2E).

**Figure S4.**
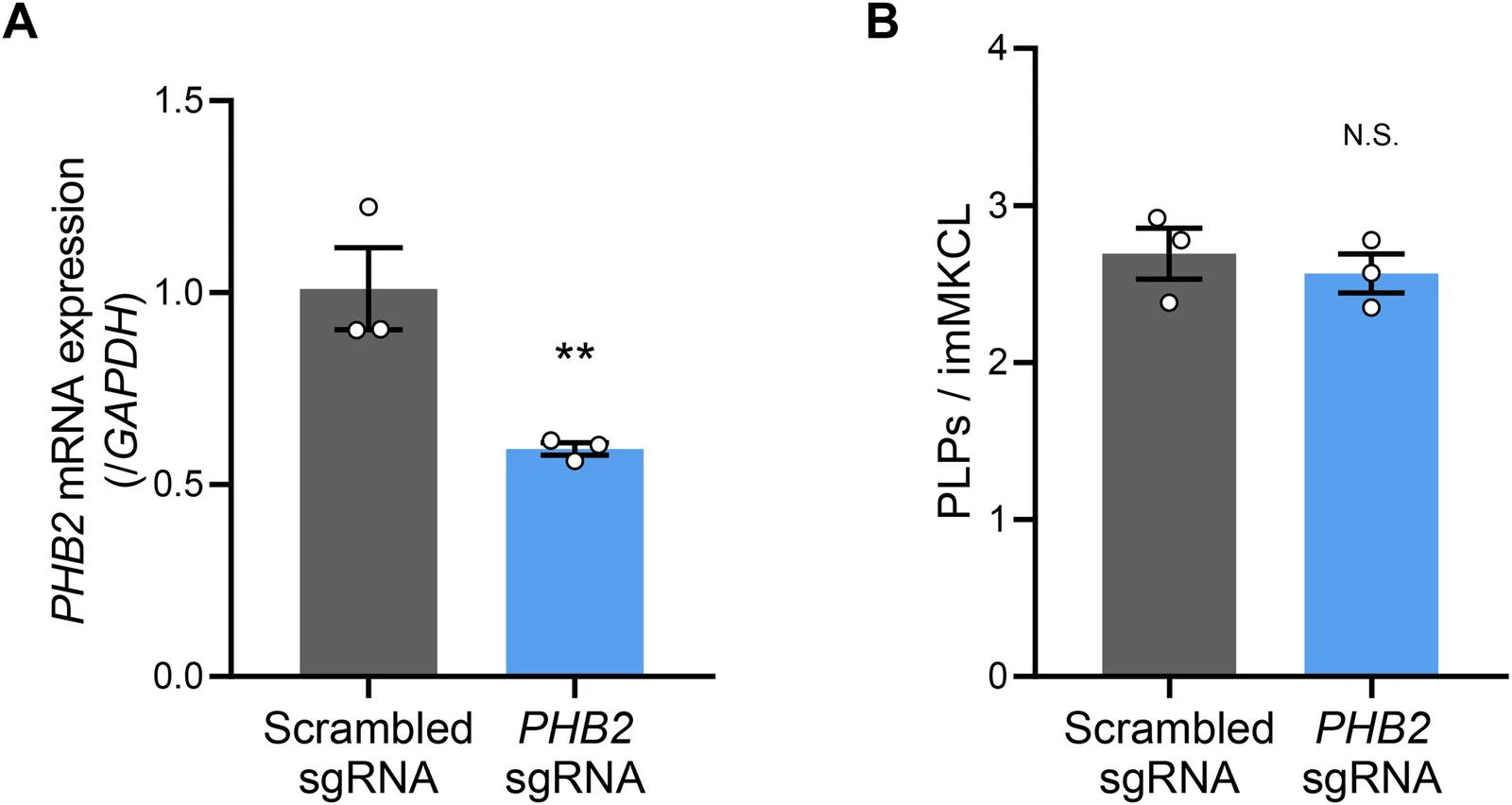
Effect of *PHB2* knockout on PLP production in the imMKCL. **(A)** Relative *PHB2* mRNA levels in wild-type and *PHB2* knockout cells cultured under DOX-OFF static conditions, measured 3 d after transfection. Data show the mean ± SEM from n = 3 independent biological replicates. Statistical comparisons were performed on ΔΔCt values using an unpaired *t* test (**P < 0.01). **(B)** Number of CD41^+^, CD42b^+^ PLPs generated from *PHB2* knockout cells in static dish culture. Data show the mean ± SEM from n = 3 independent biological replicates. Statistical comparisons were performed using an unpaired *t* test (N.S., not significant).

These findings led us to hypothesize that PHB2 suppression is critical for megakaryocyte maturation. We performed *PHB2* knockout in the imMKCL using CRISPR-Cas9 and initiated DOX-OFF induction under small-scale static culture conditions because of the limited number of transduced cells available. Although *PHB2* mRNA levels were reduced by ∼40% at Day 3, no enhancement in PLP production was observed (Fig S4A and B). Given the potential for genetic manipulation before DOX-OFF induction to compromise cellular integrity and reduce PLP production capacity, we sought to identify small-molecule compounds capable of modulating PHB2 function through simple supplementation in the culture medium.

### JI051 promotes maturation of megakaryocytes and subsequent PLP production under both static and shaking culture conditions

A small-molecule compound, designated JI051, was previously identified as a modulator of PHB2 in a study aimed at exploring pharmacological effects unrelated to platelet production (Perron et al, 2018). According to that report, JI051 is an indole-based small-molecule compound with a π-electron–rich aromatic structure and an acrylamide-like linker that interacts with PHB2 and stabilizes the interaction between PHB2 and Hes1, thereby potentially suppressing cancer cell proliferation. Because no prior studies had examined the effects of this compound in megakaryocytes, we evaluated the impact of JI051 on PLP production using the imMKCL.

We previously demonstrated that mechanical stimulation via shaking culture enhances platelet production from mature megakaryocytes (Ito et al, 2018). In the present study, we used a simplified two-dimensional shaking culture system to apply mechanical stimulation and assess the effect of JI051 on PLP yield.

**Figure 3.**
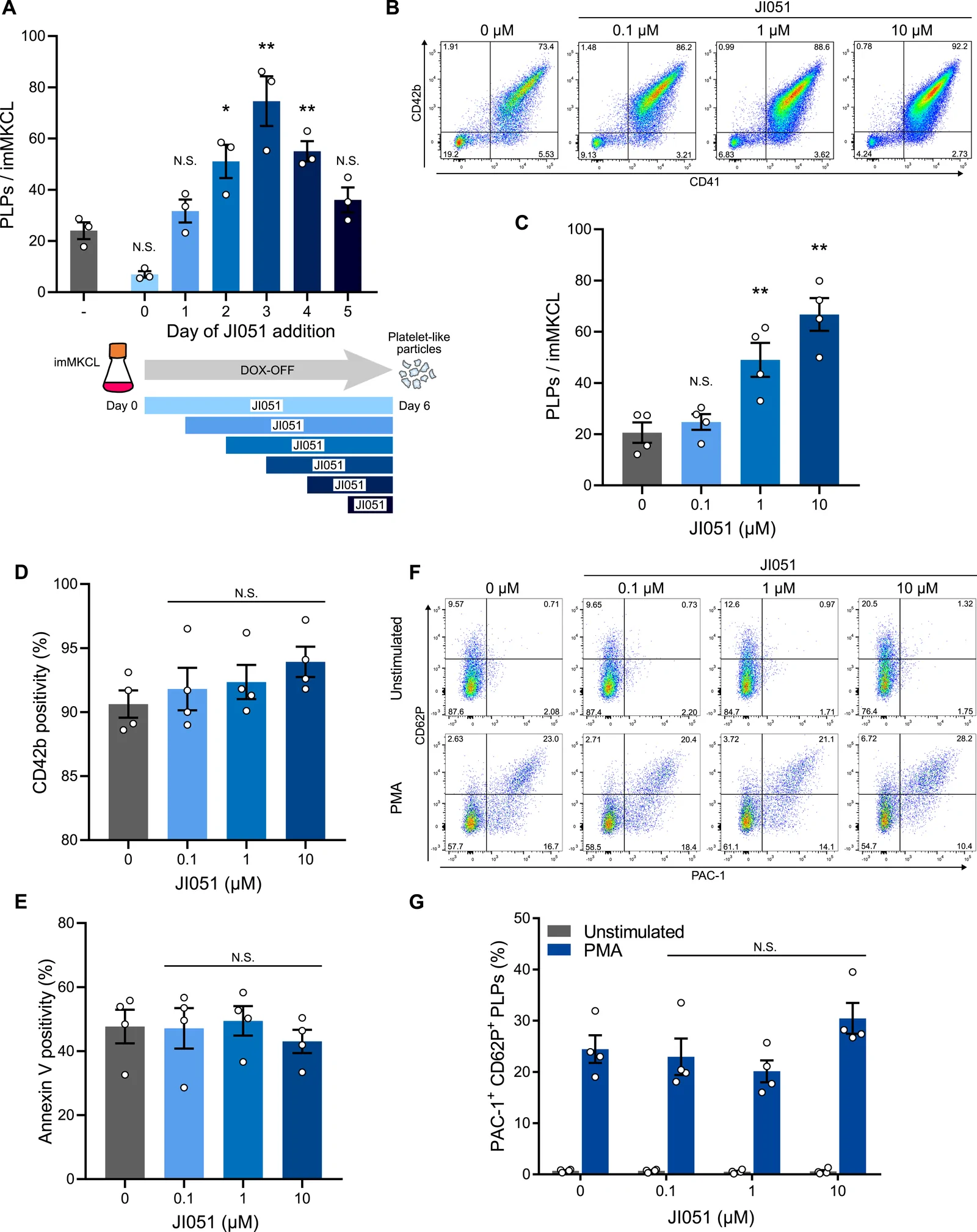
JI051, a PHB2-binding compound, enhances PLP production from the imMKCL. **(A)** Number of CD41^+^, CD42b^+^ PLPs generated from the imMKCL in flask culture upon varying the timing of 10 μM JI051 addition. Data show the mean ± SEM from n = 3 independent biological replicates. Statistical comparisons were performed versus the JI051-untreated control using Dunnett’s multiple comparisons test (*P < 0.05, **P < 0.01, N.S., not significant). **(B)** Representative dot plots from flow cytometric analysis showing CD41 and CD42b expression profiles in response to JI051 treatment ranging from 0 to 10 μM. **(C)** Number of CD41^+^, CD42b^+^ PLPs generated from the imMKCL in shaking flask culture upon varying the concentration of JI051. Data show the mean ± SEM from n = 4 independent biological replicates. Statistical comparisons were performed versus the JI051-untreated control using Williams’ test (**P < 0.01, N.S., not significant). **(D)** Percentage of CD42b^+^ PLPs generated from the imMKCL in shaking flask culture upon varying the concentration of JI051. Data show the mean ± SEM from n = 4 independent biological replicates. Statistical comparisons were performed versus the JI051-untreated control using Williams’ test (N.S., not significant). **(E)** Quantification of flow cytometric analysis of Annexin V binding on PLPs generated in shaking flask culture upon addition of JI051 at concentrations of 0–10 μM. Data show the mean ± SEM from n = 4 independent biological replicates. Statistical comparisons were performed versus the JI051-untreated control using Dunnett’s multiple comparisons test (N.S., not significant). **(F)** Representative dot plots from flow cytometric analysis showing PAC-1 and CD62P expression profiles of unstimulated and PMA-stimulated PLPs in response to JI051 treatment ranging from 0 to 10 μM. **(G)** Quantification of flow cytometric analysis of PAC-1 and CD62P epitope surface staining of unstimulated and PMA-stimulated PLPs harvested from the imMKCL in shaking flask cultures. Data show the mean ± SEM from n = 4 independent biological replicates. Statistical comparisons were performed versus the JI051-untreated control using Dunnett’s multiple comparisons test (N.S., not significant). Source data are available for this figure.

JI051 was supplemented into the culture medium at various time points from Day 0 to Day 5 of the DOX-OFF phase, and PLP production was quantified on Day 6. The addition of JI051 from Day 2 to Day 4 increased PLPs, with the largest effect observed on Day 3, resulting in an approximate threefold increase in yield (Fig 3A).

**Figure S5.**
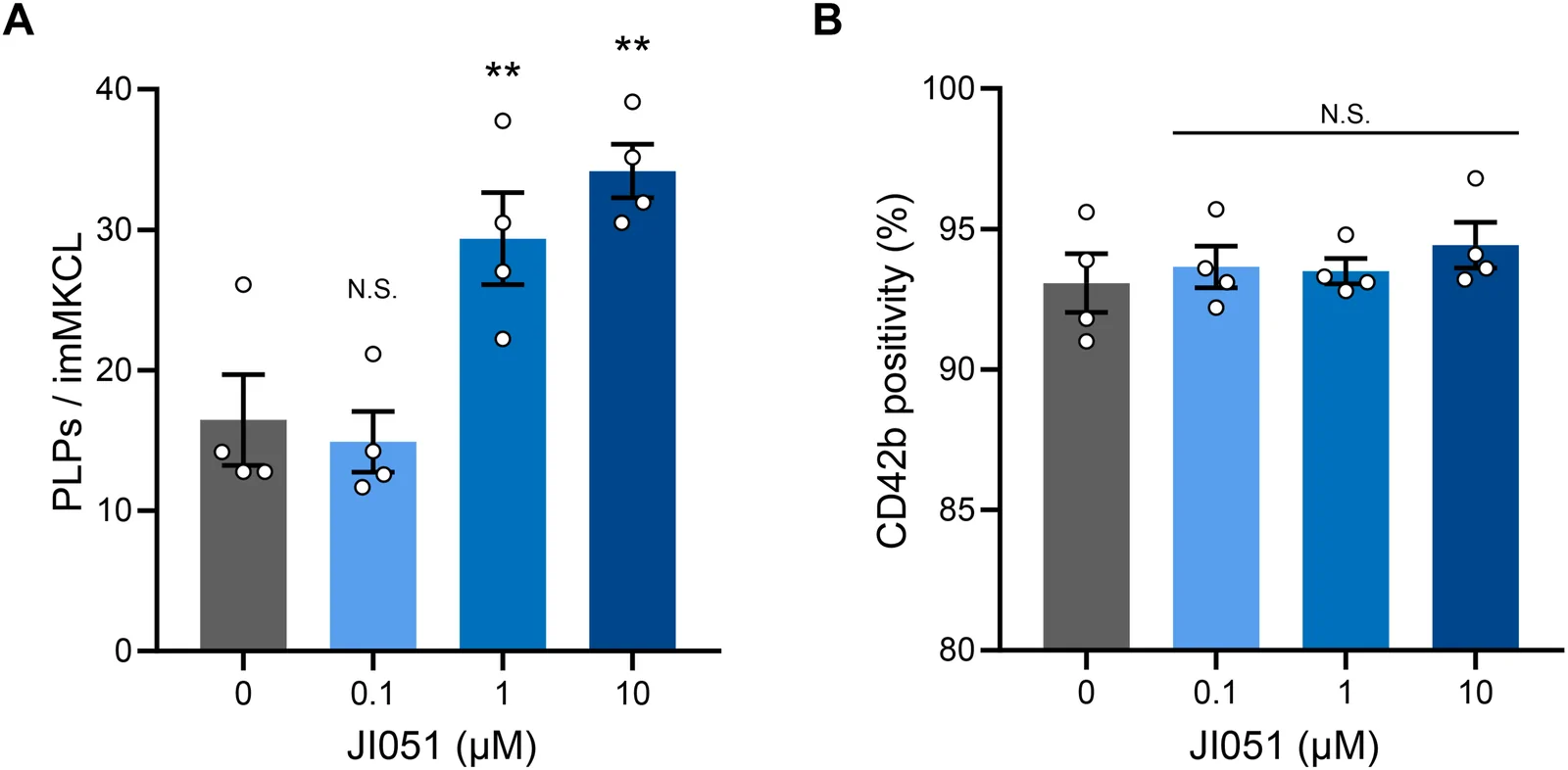
JI051 enhances PLP production under static culture conditions. **(A)** Number of CD41^+^, CD42b^+^ PLPs generated from the imMKCL in static dish culture upon varying the concentration of JI051. Data show the mean ± SEM from n = 4 independent biological replicates. Statistical comparisons were performed versus the JI051-untreated control using Williams’ test (**P < 0.01, N.S., not significant). **(B)** Percentage of CD42b^+^ PLPs generated from the imMKCL in static dish culture upon varying the concentration of JI051. Data show the mean ± SEM from n = 4 independent biological replicates. Statistical comparisons were performed versus the JI051-untreated control using Williams’ test (N.S., not significant).

We then fixed the timing of JI051 supplementation to Day 3 and investigated the optimal concentration. We evaluated three concentrations (0.1, 1, and 10 μM) to reveal a dose-dependent increase in PLP production, with the greatest effect at 10 μM among the tested doses (Fig 3B and C). This stimulatory effect was also evident under static culture conditions (Fig S5A), indicating that JI051 enhances PLP production independently of mechanical stimulation. Moreover, PLPs generated in the presence of JI051 exhibited maintained expression of CD42b, a marker of PLP quality (Robert et al, 2011; Hirata et al, 2017; Figs 3B and D, and S5B).

PLP quality was further assessed via Annexin V staining. No significant difference in Annexin V positivity was observed between JI051-treated and control groups, confirming that JI051 does not compromise PLP membrane integrity (Fig 3E). Next, the functionality of JI051-derived PLPs was assessed by stimulating cell suspensions with phorbol 12-myristate 13-acetate (PMA) and measuring activation markers PAC-1 and CD62P. The proportion of PAC-1^+^/CD62P^+^ double-positive cells, indicative of PLP activation, showed no significant difference between JI051-treated and control groups across all concentrations (Fig 3F and G). These results indicate that, although JI051 enhances PLP production, JI051-derived PLPs exhibit activation responses comparable with those of control PLPs after PMA stimulation.

**Figure S6.**
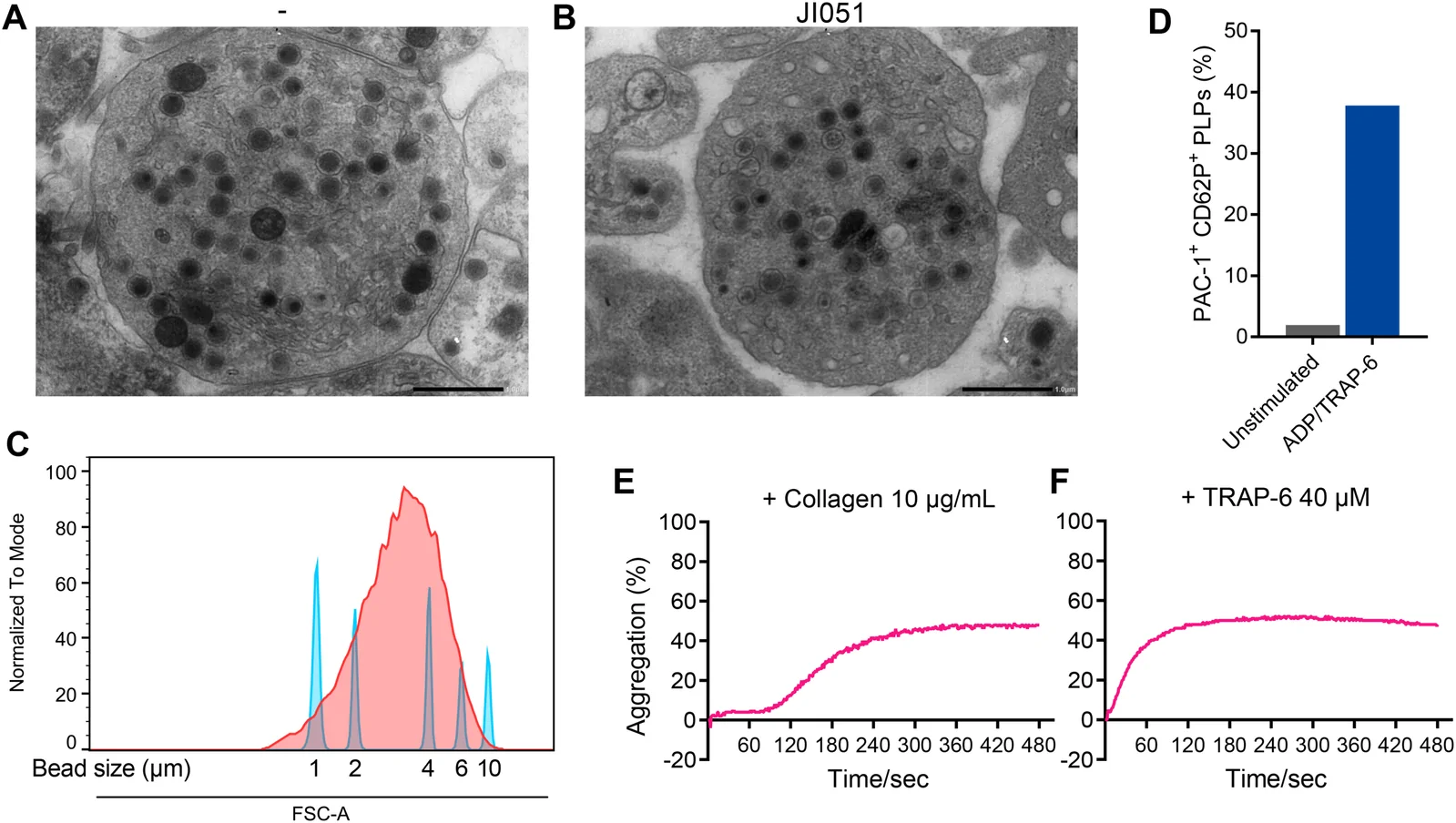
Morphological and functional properties of PLPs generated with JI051 in a liter-scale culture. **(A, B)** Representative electron microscopy images of the imMKCL-derived PLPs (A) without JI051 or (B) with JI051. Scale bar, 1 μm. Images are representative of a single liter-scale culture experiment (n = 1). **(C)** Representative FSC-A plot of the PLPs produced by JI051 (red histogram). Blue histogram indicates calibration beads of known diameters (1, 2, 4, 6, and 10 μm). **(D)** Percentage of PAC-1^+^/CD62P^+^ PLPs generated in the presence of JI051 after stimulation with 100 μM ADP and 100 μM TRAP-6. **(E, F)** Aggregation of PLPs generated with JI051 based on light transmission after agonist stimulation by (E) 10 μg/ml collagen or (F) 40 μM TRAP-6. Panels C, D, E, F show representative results from a single liter-scale culture experiment (n = 1).

To further examine the morphology and functionality of PLPs generated with JI051, we performed a liter-scale culture experiment using the VerMES vertical mixing bioreactor, as previously described (Ito et al, 2018; Okamoto et al, 2024). JI051-derived PLPs displayed granule-containing ultrastructure comparable with control PLPs (Fig S6A and B), and most ranged from 3 to 8 μm in diameter, consistent with previous reports (Ito et al, 2018; Fig S6C). Functionally, stimulation with ADP and TRAP-6 increased the proportion of PAC-1^+^/CD62P^+^ PLPs produced by JI051 (Fig S6D). These PLPs also showed aggregation in response to collagen or TRAP-6 (Fig S6E and F). Overall, in this single liter-scale culture experiment, JI051-derived PLPs retained several morphological features and exhibited basic activation responses to selected platelet agonists.

### JI051 promotes proplatelet-like extensions

**Figure 4.**
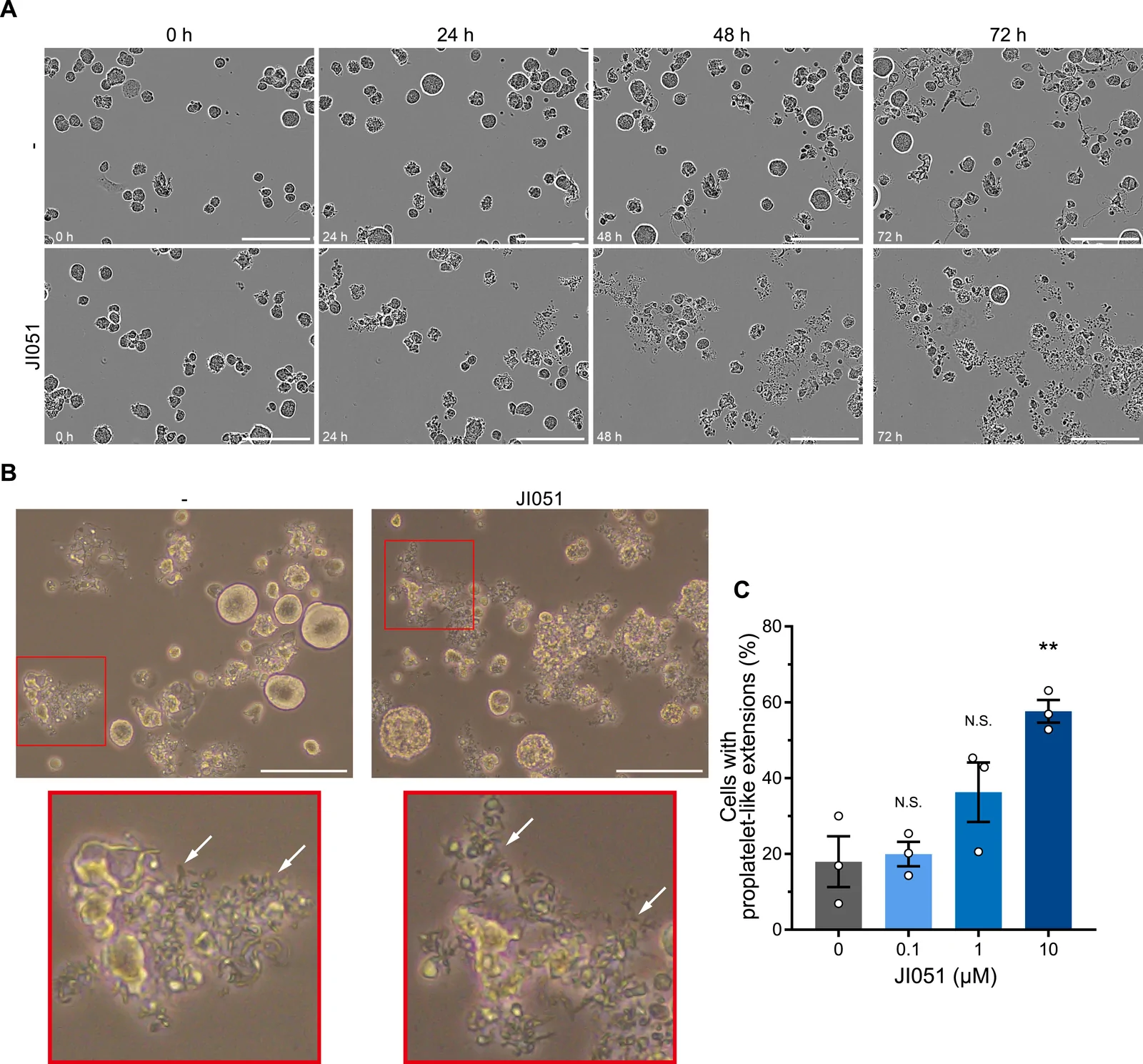
JI051 promotes proplatelet-like extensions in the imMKCL. **(A)** Time-course images of the imMKCL cells cultured with or without JI051 under static conditions in DOX-OFF culture (×20 magnification). The times indicated correspond to the elapsed time after the addition of JI051. Scale bar, 100 μm. **(B)** Representative bright-field images (×20 magnification) of the imMKCL cells cultured under static conditions on Day 6 of DOX-OFF culture. Arrows indicate proplatelet-like extensions. Scale bar, 100 μm. **(C)** Quantification of the percentage of cells harboring proplatelet-like extension in blinded images. Data show the mean ± SEM from n = 3 independent biological replicates. Statistical comparisons were performed versus the JI051-untreated control using Williams’ test (**P < 0.01, N.S., not significant). Source data are available for this figure.

Morphological changes were investigated in the imMKCL cells using IncuCyte time-lapse imaging after JI051 treatment on Day 3 of the DOX-OFF phase. Unexpectedly, no substantial cell enlargement was observed. Instead, JI051 accelerated cytoskeletal rearrangement and promoted earlier formation of proplatelet-like extensions than typically observed (Fig 4A; Video 1 and Video 2).

Video 1 Time-lapse imaging of imMKCL megakaryocytes upon DMSO treatment. The 0 h time point shown in the video indicates the timing of DMSO addition (Day 3 of the DOX-OFF maturation phase). Scale bar, 100 μm. Download video

Video 2 Time-lapse imaging of imMKCL megakaryocytes showing accelerated proplatelet-like extension upon 10 μM JI051 treatment. The 0 h time point shown in the video indicates the timing of JI051 addition (Day 3 of the DOX-OFF maturation phase). Scale bar, 100 μm. Download video

**Figure S7.**
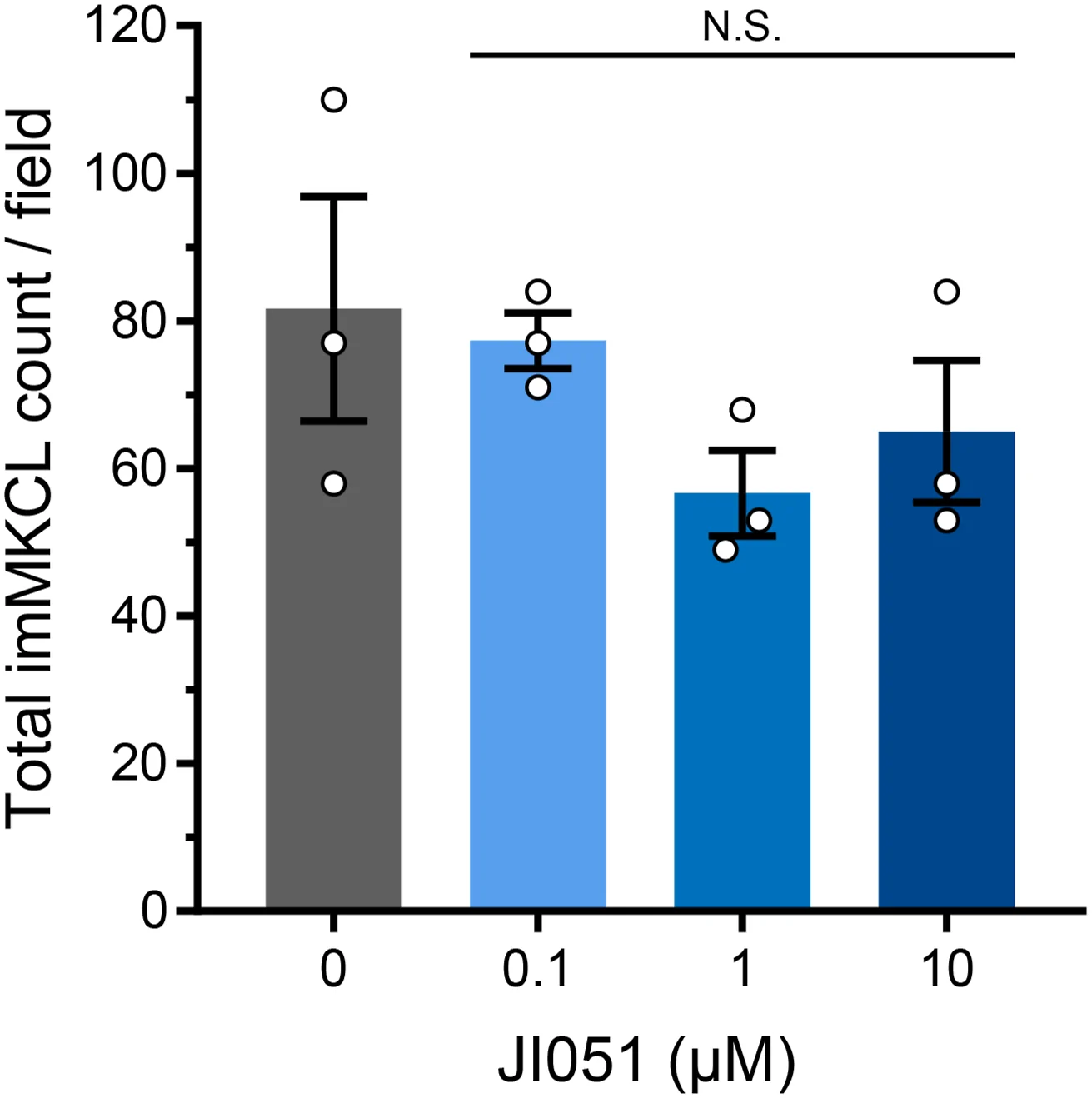
JI051 promotes proplatelet-like extensions without affecting the number of imMKCL cells. Quantification of the total number of imMKCL cells per field in Fig 4C under static conditions. Data show the mean ± SEM from n = 3 independent biological replicates. Statistical comparisons were performed versus the JI051-untreated control using Dunnett’s multiple comparisons test (N.S., not significant).

Under static culture conditions, cells were maintained in the DOX-OFF phase and were treated with JI051 at concentrations ranging from 0.1 to 10 μM on Day 3. Bright-field microscopy was performed on Day 6 to capture cell morphology. Cell membrane protrusions were identified as proplatelet-like extensions, and the proportion of cells exhibiting these structures was quantified. In the absence of JI051, ∼20% of the imMKCL megakaryocytes formed proplatelet-like extensions. JI051 treatment resulted in a dose-dependent increase in the proportion of cells with these structures. At the highest concentration (10 μM), the proportion of cells showing proplatelet-like extensions tripled to ∼60%, suggesting that a greater proportion of cells contributed to PLP production. Quantitative analysis on Day 6 confirmed a significant increase in cells exhibiting proplatelet-like extensions in the 10 μM JI051-treated group compared with the vehicle control (Fig 4B and C). Notably, the total number of imMKCL cells remained unchanged between the vehicle and JI051-treated groups (Fig S7), suggesting that JI051 specifically promotes the formation of proplatelet-like extensions in maturing megakaryocytes.

### JI051 induces gene expression changes similar to those observed during megakaryocyte maturation

Based on prior findings, we hypothesized that JI051 facilitates the terminal differentiation of imMKCL megakaryocytes into PLPs. To examine the intracellular changes induced by JI051, we conducted RNA-seq on the cells treated with or without the compound.

JI051 was supplemented on Day 3 of the DOX-OFF phase, and samples were harvested on Day 5 for RNA-seq analysis. We identified 4,446 differentially expressed genes (DEGs), which were analyzed using the BaseSpace Correlation Engine platform to evaluate their associations with publicly available datasets.

**Figure 5.**
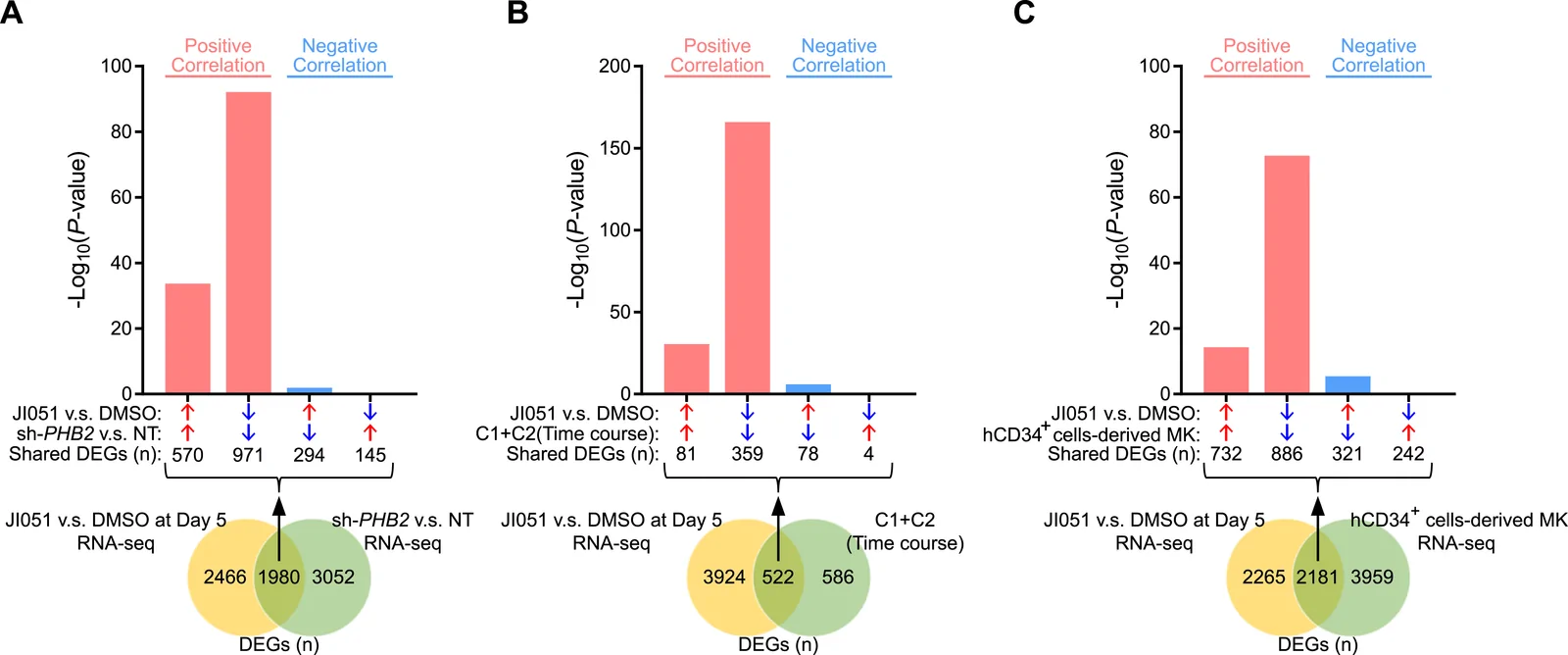
JI051 induces transcriptional changes in the imMKCL resembling megakaryocyte maturation patterns. **(A, B, C)** Correlation plots comparing the JI051-supplemented imMKCL RNA-seq dataset with (A) the *PHB2* knockdown dataset, (B) our proteomics C1+C2 dataset (culture duration), and (C) a megakaryocyte maturation dataset. DEGs, differentially expressed genes; MK, megakaryocyte; NT, non-targeting shRNA.

First, we compared JI051-induced gene expression changes with those observed in *PHB2* knockdown datasets identified through our proteomic analysis. Of the 1,980 overlapping DEGs, 1,541 (570 + 971) exhibited similar expression patterns, indicating a significant positive correlation (Fig 5A and Table S5). These findings suggest that JI051 addition during the maturation phase induces transcriptional changes analogous to those triggered by *PHB2* knockdown.

Table S5. Common genes between the JI051 Day 5 DEG dataset and the *PHB2* knockdown dataset.

Next, we compared JI051-induced transcriptional changes with our proteomic dataset. These changes showed a positive correlation with protein groups C1 and C2, which reflected temporal transitions from Day 3 to Day 6 during the DOX-OFF phase (Fig 5B and Table S6).

Table S6. Common genes between the JI051 Day 5 DEG dataset and the C1+C2 dataset.

Furthermore, JI051-induced gene expression profiles correlated with temporal transcriptional changes observed during the differentiation and maturation of human CD34^+^ cells into megakaryocytes (Mallo et al, 2021 *Preprint*) (Fig 5C and Table S7).

Table S7. Common genes between the JI051 Day 5 DEG dataset and a megakaryocyte maturation dataset.

Collectively, these results suggest that JI051 induces transcriptional changes that promote megakaryocyte maturation, thereby contributing to enhanced PLP production.

## Discussion

Megakaryocytes constitute a heterogeneous cell population, exhibiting variability in morphology, polyploidization, and platelet production capacity. Even when derived in vitro from hematopoietic stem cells, megakaryocytes display substantial heterogeneity, with only a fraction contributing to platelet production (Bornstein et al, 2001; Mattia et al, 2002). Our group has focused on the clinical application of artificial platelets generated from imMKCL megakaryocytes (Sugimoto et al, 2022a, 2022b). However, even these cells retain a certain degree of intrinsic heterogeneity, necessitating the identification and enrichment of subpopulations with high platelet-producing potential.

Here, we established a methodology to identify candidate regulatory factors by analyzing proteomic differences among imMKCL subpopulations at distinct maturation stages. Using the BaseSpace Correlation Engine platform, we identified candidate genes potentially involved in regulating the transition from immature to mature megakaryocytes. This approach led to the identification of the small molecule JI051, which significantly enhanced PLP production from imMKCL cells, achieving a threefold increase in yield. This enabled the production of ∼10^11^ PLPs in a 2–3 liter culture system, effectively reducing manufacturing costs by nearly two-thirds. Importantly, JI051 can be adequately removed during final purification to comply with the guidelines of the International Council for Harmonisation, and we have also developed new compounds with enhanced potency at lower concentrations. These findings mark an advance toward the industrial-scale manufacture of artificial platelets.

We initially compared protein expression profiles between enlarged and non-enlarged imMKCL cells under standard DOX-OFF conditions. Because more than 90% of proteins showed less than a twofold change or lacked statistical significance, making it challenging to identify individual targets, we shifted our focus to upstream regulatory factors. PCA delineated two major axes: PC1 (temporal) and PC2 (size), and classified proteins into four groups (C1–C4). Interrogation of these lists using omics databases via Correlation Engine identified seven candidate genes: *PAN2*, *PHB2*, *ATP6V0B*, *TNFRSF21*, *GNAS*, *ARG1*, and *AKR1A1*. Among these, *PHB2* is noteworthy because of its reported role in hematopoietic stem cell function (Liu et al, 2017), and its interaction with JI051 guided the discovery of the compound’s PLP-enhancing effect (Fig 3). Rather than relying on a simple two-group comparison, we used PCA to extract protein groups with strong contributions to maturation, which we regard as critical for uncovering these insights.

Morphological analyses suggested that JI051 promotes proplatelet-like extensions. Previous studies have reported that proplatelet formation is associated with changes in cell cycle status and centrosome clustering (Becker et al, 2024). Given that JI051 induces cell cycle arrest in cancer cells (Perron et al, 2018), it may similarly influence the cell cycle during megakaryocyte maturation. Supporting this possibility, our independent compound screening study identified microtubule polymerization inhibitors, known to perturb the cell cycle, as enhancers of platelet production from imMKCL cells (Nakamura et al, 2025). Furthermore, RNA-seq analysis revealed that JI051 induces transcriptional changes resembling those observed during the differentiation and maturation of CD34^+^ cells into megakaryocytes, indicating that JI051 may contribute to transcriptional regulation.

Our proteomic analysis identified JI051 as a promising additive for the cell culture process. However, the functional role of its putative binding partner, PHB2, in megakaryocyte maturation and PLP biogenesis remains unresolved. Although *PHB2* expression was found to decrease during maturation, its genetic knockout induced cellular toxicity, and targeted inhibition at DOX-OFF Day 3 was not feasible, preventing accurate assessment of its impact on PLP production. Further analyses, including subcellular localization studies and the use of PHB2-targeting heterobifunctional degraders, may provide additional insight into the role of PHB2 in megakaryocyte development. A previous report indicated that JI051 interacts not only with PHB2 but also with Hes1 (Perron et al, 2018); however, *Hes1* expression in megakaryocytes is minimal, and its contribution to the mechanism of action in this context remains to be clarified. Therefore, further studies are needed to elucidate the precise molecular mechanism by which JI051 promotes PLP biogenesis, and further improvement in PLP yield for clinical-grade manufacturing may be achievable once the mechanism of action is elucidated. In addition, neither JI051 supplementation nor *PHB2* knockdown contributed to the promotion of megakaryocyte enlargement. These findings imply that JI051 facilitates PLP production independently of typical markers of megakaryocyte maturation, resembling the characteristics of human fetal megakaryocytes, which are smaller and exhibit lower levels of polyploidy than adult cells (Sola-Visner et al, 2007; Nakamura et al, 2025). Further investigation is warranted to determine whether other knockdown candidate factors or their associated compounds identified in this study influence megakaryocyte polyploidization. Discovery of such compounds may further enhance PLP production efficiency when used in combination with JI051.

In conclusion, we developed a methodology to detect subtle differences among phenotypically similar cell populations and to identify candidate factors for phenotype conversion. This approach may serve as a valuable tool for dissecting and managing heterogeneity in cell culture systems. Ultimately, the identification of JI051 as a PLP production enhancer represents an advance in manufacturing and quality control for artificial platelet production. The use of this compound is expected to facilitate the development of artificial platelet concentrates for emergency preparedness and foster sustainable manufacturing practices.

## Materials and Methods

### Cell preparation and culture

An imMKCL (clone 7) was generated from human iPSCs using DOX-inducible transcription factors, as previously described (Nakamura et al, 2014; Ito et al, 2018).

#### DOX-ON proliferation culture

Expansion of the imMKCL under DOX-ON conditions was performed according to established protocols (Ito et al, 2018). Cells were cultured in a humidified incubator at 37°C and 5% CO_2_ and passaged to maintain a density of 1 × 10^5^ to 1 × 10^6^ cells/ml.

#### DOX-OFF differentiation culture

For PLP production after induction of imMKCL maturation (DOX-OFF stage), ∼1 × 10^5^ cells/ml were cultured either in static plates or in 125-ml Erlenmeyer cell culture flasks (catalog No. 431143; Corning) under orbital shaking conditions (100 rpm) using a Lab-Therm shaker (Kuhner) or a New Brunswick S41i incubator (Eppendorf). A 10 mM stock solution of JI051 in dimethyl sulfoxide (DMSO) was diluted in culture medium as needed and added on Day 3 or at the indicated time points.

For morphological and functional assessment, PLPs were produced by culturing imMKCL cells at a scale of 1.2 or 2.4 liters for 6 d in a VerMES vertical mixing bioreactor (SATAKE MultiMix), as previously described (Ito et al, 2018; Sugimoto et al, 2022b; Okamoto et al, 2024). JI051 was added on Day 3. The PLP-containing culture medium was concentrated and purified using a hollow-fiber cylinder and an ACP215 cell centrifugation system (Haemonetics). The resulting PLPs were resuspended in bicarbonated Ringer’s solution supplemented with 10% acid citrate dextrose solution A and 2.5% albumin.

### IncuCyte imaging system

imMKCL cells at the DOX-OFF stage were seeded into 24-well plates and imaged using the IncuCyte S3 system (Sartorius). Images were acquired every hour from four distinct regions per well using a ×20 objective lens. At 65 h after culture initiation, 10 μM JI051 was added to each well, and the plates were returned to the IncuCyte for continuous imaging. Cultures were maintained at 37°C in a humidified atmosphere containing 5% CO_2_. The acquired data were analyzed using Fiji (ImageJ) version 1.54p (National Institutes of Health).

### Single-guide RNA transduction

Knockout of *PHB2* in the imMKCL was performed using a 4D-Nucleofector system (Lonza) according to the manufacturer’s instructions. Three single-guide RNAs (sgRNAs) targeting *PHB2* or three non-targeting control sgRNAs (Alt-R CRISPR-Cas9 sgRNA; Integrated DNA Technologies; sequences shown in Table S8) were mixed at equimolar amounts and gently pipetted with Cas9 protein solution (catalog No. A36497; Thermo Fisher Scientific). Cells were washed with Dulbecco’s phosphate-buffered saline and temporarily resuspended in DOX-OFF medium. After centrifugation, the cells were resuspended in Buffer P3 (catalog No. V4XP-3032; 4D-Nucleofector P3 Primary Cell Kit, Lonza) and combined with the Cas9/sgRNA mixture. Electroporation was carried out using program CA-137 on the 4D-Nucleofector system. The cells were then resuspended in DOX-OFF medium and seeded into 48-well plates.

Table S8. Protospacer sequences of sgRNAs used in this study.

### Flow cytometry

Flow cytometric analysis of PLPs was performed as previously described (Nakamura et al, 2014, 2025; Ito et al, 2018). On the DOX-OFF stage, 100 μl of cell suspension was collected and mixed with Trucount beads (catalog No. 340334; Becton, Dickinson and Company [BD]). A cocktail of fluorophore-conjugated antibodies (CD41-APC, 1:40, catalog No. 303710; CD42b-PE, 1:40, catalog No. 303906; BioLegend) was added, and samples were incubated for 20 min at room temperature in the dark. After the addition of 400 μl HEPES-Tyrode buffer, stained PLPs and Trucount beads were quantified by FACS Verse (BD) or LSR Fortessa flow cytometer (BD). At least 5,000 Trucount bead events or 180 s were acquired per sample. Sequential gating for identifying PLPs was applied as follows: debris and residual imMKCL cells were excluded based on FSC/SSC profiles or DRAQ5 (catalog No. DR50200; BioStatus) positivity if needed, followed by singlet discrimination using FSC-H versus FSC-W and SSC-H versus SSC-W. PLPs were quantified as CD41-APC and CD42b-PE double-positive events based on unstained controls. Absolute PLP numbers per 100 μl of cell suspension were calculated as (CD41^+^/CD42b^+^ PLP events)/(Trucount events) × (total number of Trucount beads in each Trucount tube). Data were analyzed with FlowJo software (BD, version 10).

For PLP activation analysis, PMA (catalog No. 162-23591; FUJIFILM Wako Pure Chemical), ADP (catalog No. A4386; Sigma-Aldrich), and TRAP-6 (catalog No. 3497/5; Tocris Bioscience) were used as platelet agonists. A 200-μl aliquot of cell suspension was incubated with fluorophore-conjugated antibodies in the presence of PMA (final concentration: 400 nM) or a combination of ADP and TRAP-6 (final concentration: 100 μM each) for 30 min at room temperature in the dark. After the addition of 400 μl of HEPES-Tyrode buffer, PAC-1-FITC (catalog No. 340507; BD) and CD62P-APC (catalog No. 304910; BioLegend) double-positive PLP events were quantified as activated PLPs by flow cytometry.

For apoptosis analysis, 200 μl of cell suspension was incubated with either HEPES-Tyrode buffer or ionomycin (final concentration: 20 μM) for 30 min at room temperature. FITC-conjugated Annexin V (catalog No. 556419; BD) was then added, and Annexin V-positive PLP events were quantified by flow cytometry.

For PLP size distribution assay, the Flow Cytometry Size Calibration Kit (catalog No. F13838; Thermo Fisher Scientific) was added to samples together with anti-CD41 and anti-CD42b antibodies. FSC-A distributions of CD41^+^/CD42b^+^ double-positive PLPs and calibration beads with diameters of 1, 2, 4, 6, and 10 μm were displayed as overlaid histograms.

### Quantitative RT–PCR

Total RNA was extracted from 1 × 10^5^ cells or 1 ml of cell suspension using the Maxwell RSC simplyRNA Cells Kit (catalog No. AS1390; Promega), according to the manufacturer’s instructions. cDNA was synthesized from 120 ng total RNA using the ReverTra Ace qPCR RT Master Mix (catalog No. FSQ-301; Toyobo). For the *PHB2* knockout study, RNA extraction and quantitative RT–PCR were performed using the Cells-to-C_T_ 1-Step TaqMan Kit (catalog No. A25603; Thermo Fisher Scientific) in sgRNA-transduced cells. Quantitative RT–PCR was carried out using a 7500 Fast Real-Time PCR system (Thermo Fisher Scientific). The following TaqMan probes were used: *PHB2* (Hs00200720_m1) and *GAPDH* (Hs99999905_m1) (Thermo Fisher Scientific). Relative gene expression levels were normalized to *GAPDH* and calculated using the 2^−ΔΔCt^ method.

### Sample preparation for proteome analysis

The imMKCL megakaryocytes were cultured under DOX-OFF conditions, and the cells were then centrifuged at 50*g* for 2 min at room temperature. Both the supernatant and pellet were collected. The supernatant was further centrifuged at 200*g* for 5 min at room temperature, and the resulting pellet was collected. Pellets from both centrifugation steps were combined and resuspended in HEPES-Tyrode buffer, followed by staining with CD41-APC and CD42b-PE antibodies.

The stained cell suspension was subjected to flow cytometry using a FACSAria III (BD). Based on the FSC-A histogram of Day 3, cells corresponding to the top, middle, and bottom 10% of the CD41^Hi^/CD42b^Hi^ population were sorted (Fig S1A). The gating strategy was kept identical for the Day 6 samples. Sorted cells were centrifuged at 200*g* for 5 min at 4°C, and the resulting pellets were incubated on ice for 20 min in 25 μl of Lysis Buffer A (10 mM HEPES-KOH, pH 7.9, 1 mM EDTA, 10 mM KCl, 1 mM dithiothreitol, and protease inhibitors). Subsequently, 1.6 μl of 10% NP-40 solution was added, the mixture was vortexed for 15 s, and centrifuged at 16,000*g* for 30 s at 4°C. The supernatant was collected as the cytoplasmic fraction.

The remaining pellet was resuspended in 10 μl of Lysis Buffer B (20 mM HEPES-KOH, pH 7.9, 400 mM NaCl, 1 mM EDTA, 1 mM EGTA, and protease inhibitors), vortexed, and incubated on ice for 30 min. After centrifugation at 14,000*g* for 5 min at 4°C, the supernatant was collected as the nuclear fraction.

Protein concentration was determined using the EZQ Protein Quantitation Kit (catalog No. R33200; Thermo Fisher Scientific). A volume equivalent to 5 μg of protein was transferred to a 96-well plate and adjusted to 20 μl with Milli-Q water. To this, 1.5 μl of 100 mM dithiothreitol was added and the mixture was incubated at 70°C for 15 min. Subsequently, 2 μl of 500 mM iodoacetamide was added and incubated in the dark at room temperature for 30 min.

SP3 beads were added, and desalting and purification were performed using the BRAVO automated system (Agilent Technologies). Proteins were digested overnight at 37°C with a trypsin-LysC solution. The resulting peptides were desalted and purified using standard procedures and subjected to mass spectrometry analysis.

### LC-MS analysis and data processing

After tryptic digestion, peptide samples were analyzed by liquid chromatography–mass spectrometry (LC-MS). For each run, 3 μg of peptide mixture was injected into an Ultimate 3000 nanoUHPLC system (Thermo Fisher Scientific) coupled to an Orbitrap Fusion mass spectrometer (Thermo Fisher Scientific). Peptides were loaded onto a trap column (Acclaim PepMap100, catalog No. 164564; Thermo Fisher Scientific) in buffer A (0.1% formic acid) and separated on an analytical column (nanoESI column, 15 cm × 75 μm, NTCC-360/75-3-155; Nikkyo Technos) maintained at 35°C. Separation was performed at 300 nl/min using a linear gradient from 4% to 40% buffer B (100% acetonitrile, 0.1% formic acid) over 100 min, followed by a 1-min increase to 90% buffer B and a 5-min wash at 90% buffer B.

Mass spectrometry was performed using data-dependent acquisition. Detailed MS acquisition parameters are summarized in Table S9. Raw data were processed using Proteome Discoverer Software 2.5 (Thermo Fisher Scientific) against the UniProt human proteome database (UP000005640). Peptide-spectrum matches were validated using Percolator, and protein identifications were filtered at a false discovery rate of <1%. Label-free quantification was performed based on precursor ion intensities.

Table S9. Detailed MS acquisition parameters.

### Data analysis

For selected analyses, we used the R statistical computing environment (R Foundation for Statistical Computing) along with established packages (Table S10). Pathway enrichment analyses were performed using standard GO terms (Mi et al, 2019). Enrichment plots were generated to highlight the most significantly enriched pathways. To identify candidate target genes capable of promoting imMKCL maturation, the BaseSpace Correlation Engine platform (previously known as NextBio) was used, along with the Knockdown Atlas tool (Illumina) (Kupershmidt et al, 2010).

Table S10. R environment for proteomics analysis.

### Quantification of proplatelet-like extensions

Proplatelet-like extensions were quantified using bright-field microscopy images captured with a CKX53 inverted microscope (Olympus) using a ×20 objective lens. Cells were imaged on Day 6 of the DOX-OFF phase. Approximately 50 cells located at the center of each well were imaged and analyzed per condition and per replicate. The number of cells exhibiting proplatelet-like extensions was counted under blinded conditions, and the proportion of proplatelet-like extension-positive cells was calculated relative to the total number of imMKCL cells analyzed.

### RNA sequencing

Under shaking culture conditions, imMKCL cells were treated with either 10 μM JI051 or an equivalent volume of DMSO on Day 3 of the DOX-OFF phase. Cells were harvested on Day 5, and total RNA was extracted using the RNeasy Micro Plus Kit (catalog No. 74034; Qiagen). RNA-seq library preparation was performed as previously described (Chen et al, 2024), and sequencing was conducted as 60-bp single-end reads on a HiSeq 2500 (Illumina). TopHat (version 2.1.1, with default parameters) was used to map reads to the reference genome (UCSC/hg19) with annotation data from iGenomes (Illumina). Gene expression levels were quantified as fragments per kilobase of exon per million mapped sequence reads (FPKM) using Cuffnorm (Cufflinks version 2.2.1; --output-format cuffdiff). Because biological replicates were not available (n = 1 per condition), DEGs were defined using the following criteria: sum (FPKM) ≥ 1 across the two samples, an absolute FPKM difference ≥ 1, and an absolute log_2_ fold change ≥ 0.5 (FPKM-based), yielding 4,446 DEGs.

### PLP aggregation assay

PLP aggregation was measured for light transmission using an aggregometer (MCM HEMA TRACER 313 M Model PAM-12C; LMS), as previously described (Ito et al, 2018; Okamoto et al, 2024). PLPs were resuspended in 70% human plasma (3 × 10^8^ PLPs/ml) with 2 mM CaCl_2_. A 200-μl aliquot of the sample preparation was stimulated with collagen (final concentration: 10 μg/ml) or TRAP-6 (final concentration: 40 μM) for 8 min at 37°C.

### Electron microscopy

PLPs were collected by centrifugation at 1,200*g* for 5 min and pre-fixed overnight with 2% paraformaldehyde and 0.5% glutaraldehyde. Samples were post-fixed with 1% osmium tetroxide, stained en bloc with uranyl acetate, dehydrated through a graded ethanol series, embedded in epoxy resin, and processed for ultrathin sectioning. Sections were stained with lead citrate and examined by transmission electron microscopy (JEM-1400Plus; JEOL).

### Statistical analyses

Data are expressed as the mean ± standard error of the mean (SEM) from at least three independent experiments, unless otherwise specified. For comparisons between two groups, an unpaired *t* test was used. For quantitative RT–PCR data, statistical comparisons were performed on ΔΔCt values before exponential transformation, as 2^−ΔΔCt^ values are not normally distributed. For comparisons of multiple treatment groups with a single control group, a Dunnett’s multiple comparisons test was used based on a one-way analysis of variance. For dose–response studies assessing prespecified monotonic trends, a Williams’ test was applied in the prespecified direction, with significance evaluated at a one-sided α = 0.025 to maintain an overall two-sided significance level of α = 0.05. Statistical analyses were performed using SAS software (release 9.4; SAS Institute). A two-sided significance level of α = 0.05 was considered statistically significant. Graphs were generated using GraphPad Prism version 7 (GraphPad Software).

### Declaration of Generative AI and AI-assisted Technologies in the Writing Process

During the preparation of this manuscript, the authors used Microsoft Copilot to improve language and readability, with caution. After using this tool/service, the authors reviewed and edited the content as needed, and take full responsibility for the content of the publication.

## Supplementary Material

Reviewer comments

## Data Availability

Mass spectrometry proteomics data have been deposited with the ProteomeXchange Consortium via the jPOST partner repository under the dataset identifier PXD082807. The raw RNA-seq data have been deposited in the NCBI Sequence Read Archive under BioProject accession PRJNA1516462, and the corresponding processed data have been deposited in the NCBI Gene Expression Omnibus under accession GSE344863. The data supporting the findings of this study are available from the corresponding author upon reasonable request.
