# Supplementary material for: Proteomics of heterogeneous megakaryocytes identifies JI051 as an enhancer of platelet biogenesis
Source: Life Sci Alliance. 2026 Sep 23;9(12):e202603636. doi: 10.26508/lsa.202603636 (PMC13601833; doi:10.26508/lsa.202603636)
Supplement: Supplementary file 1 [file LSA-2026-03636_review_history.pdf]

Proteomics of heterogeneous megakaryocytes identifies JI051 as an enhancer of platelet biogenesis

Ryosuke Taga, Nozomi Kuwano, Yasuo Harada, Takashi Hayashi, Yuki Sakamoto, Kosuke Fujio, Yusuke Kakumoto, Emiri Nakamura, Koji Eto, and Hideki Hayashi
DOI: https://doi.org/10.26508/lsa.202603636

Corresponding author(s): Hideki Hayashi, Otsuka

Review Timeline:

Submission Date:	2026-01-23
Editorial Decision:	2026-03-12
Revision Received:	2026-06-12
Editorial Decision:	2026-07-27
Revision Received:	2026-09-08
Accepted:	2026-09-08

Scientific Editor: Tim Fessenden

Transaction Report:

(Note: With the exception of the correction of typographical or spelling errors that could be a source of ambiguity, letters and reports are not edited. The original formatting of letters and referee reports may not be reflected in this compilation.)

March 12, 2026

Re: Life Science Alliance manuscript #LSA-2026-03636

Dr. Hideki Hayashi
Otsuka
Department of Open Innovation, Tokushima Research Center for Drug Discovery, Pharmaceutical Division
463-10 Kagasuno, Kawauchi-cho
Tokushima, Tokushima 771-0192
Japan

Dear Dr. Hayashi,

Thank you for submitting your manuscript entitled "Proteomics of heterogeneous megakaryocytes reveals JI051 as a novel enhancer of platelet biogenesis" to Life Science Alliance. The manuscript was assessed by expert reviewers, whose comments are appended to this letter.

As you will see, reviewers found the work intriguing and appreciated the potential to guide a new approach for platelet generation ex vivo. That said, all reviewers expressed important concerns on methodology, validation, and important claims made that must be resolved in a revised manuscript. Namely, a suitable revision must address all major points noted by Reviewer 1, points 1-3 by Reviewer 2, and point 1 by Reviewer 3. These comments cover essential details on methodology, controls, and central claims on demarcation membrane system formation, platelet function, and proplatelet formation. We concur this work would also be improved by addressing point 3 by Reviewer 3. While your rebuttal must address all reviewer points in some form, additional data beyond those points mentioned above are not required in a revision.

I would be happy to discuss the revision in more detail via email or phone/videoconferencing. Please let me know which option you prefer, if any.

To upload the revised version of your manuscript, please log in to your account: https://lsa.msubmit.net/cgi-bin/main.plex

You will be guided to complete the submission of your revised manuscript and to fill in all necessary information. Please get in touch in case you do not know or remember your login name.

The typical timeframe for revisions is three months. Please note that papers are generally considered through only one revision cycle, so strong support from the referees on the revised version is needed for acceptance.

While you are revising your manuscript, please also attend to the below editorial points to help expedite the publication of your manuscript. Please direct any editorial questions to the journal office. When submitting the revision, please include a letter addressing the reviewers' comments point by point.

Thank you for this interesting contribution to Life Science Alliance. We hope that the comments below will prove constructive as your work progresses, and we are looking forward to receiving your revised manuscript.

Sincerely,

Tim Fessenden
Scientific Editor
Life Science Alliance

A. THESE ITEMS ARE REQUIRED FOR REVISIONS

-- A letter addressing the reviewers' comments point by point.

-- An editable version of the final text (.DOC or .DOCX) is needed for copyediting (no PDFs).

-- High-resolution figure, supplementary figure and video files uploaded as individual files: See our detailed guidelines for preparing your production-ready images, https://www.life-science-alliance.org/authors

-- Summary blurb (enter in submission system): A short text summarizing in a single sentence the study (max. 200 characters including spaces). This text is used in conjunction with the titles of papers, hence should be informative and complementary to the title and running title. It should describe the context and significance of the findings for a general readership; it should be written in the present tense and refer to the work in the third person. Author names should not be mentioned.

-- By submitting a revision, you attest that you are aware of our payment policies found here: https://www.life-science-alliance.org/copyright-license-fee

B. MANUSCRIPT ORGANIZATION AND FORMATTING:

Full guidelines are available on our Instructions for Authors page, https://www.life-science-alliance.org/authors

We encourage our authors to provide original source data, particularly uncropped/-processed electrophoretic blots and spreadsheets for the main figures of the manuscript. If you would like to add source data, we would welcome one PDF/Excel-file per figure for this information. These files will be linked as supplementary "Source Data" files.

IMPORTANT: It is Life Science Alliance policy that if requested, original data images must be made available. Failure to provide original images upon request will result in unavoidable delays in publication. Please ensure that you have access to all original microscopy and blot data images before submitting your revision.

Reviewer #1 (Comments to the Authors (Required)):

In their manuscript, Taga et al. address an important bottleneck for ex vivo platelet manufacturing from iPSC-derived immortalized megakaryocyte cell lines (imMKCLs), namely cellular heterogeneity that limits efficient maturation and platelet release. By combining size-stratified proteomics with principal component analysis, the authors identify prohibitin-2 (PHB2) as a putative inhibitory node and test JI051 (reported to interact with PHB2) as a simple culture additive. The authors report that JI051 added during maturation accelerates proplatelet formation and increases platelet-like particle yield (approximately three-fold under optimal timing/dose). Overall, identifying JI051 as a potential modulator that increases platelet output in imMKCLs is interesting and could have clinical relevance. However, the current manuscript lacks critical methodological detail and a sufficiently rigorous phenotypic/functional characterization to support the strength of the claims. While a deep mechanistic dissection may be beyond scope, a more comprehensive characterization of the observed phenotype is required prior to publication.

Major comments

1. Definition and validation of "cell size fractions" line 102): Please report the mean actual cell size (diameter) for each fraction and include representative images of the sorted cells. It is unclear whether the gating strategy was kept identical between the two timepoints or adjusted to capture the top/bottom 10% at each timepoint independently. Please clarify explicitly and expand the Methods to describe the gating strategy in sufficient detail for reproducibility.

2. Claims about demarcation membrane system (DMS) are not supported in this manuscript (line 146). The statement that large cells at Day 6 exhibit well-developed demarcation membrane systems indicative of readiness for platelet release is not demonstrated in the current work. If this conclusion is based on prior publications, please cite them clearly. If it is meant as an observation from this study, then representative ultrastructural evidence (or appropriate imaging) should be included.

3. Incomplete reporting and statistics in Fig. 3. The JI051 concentration used in the time-series experiment is not specified (please report in the figure legend). Statistical testing is missing for Fig. 3A. Please include appropriate tests. Dose-response characterization is incomplete (Fig. 3C).

The effect of concentrations >10 µM is not shown. Does the platelet-like particle yield plateau, continue to increase, or decline (toxicity/off-target effects)?

4. Platelet-like particle benchmarking and functional assessment are currently insufficient (Fig. 3D-G).

- Please include freshly isolated platelets as an additional benchmark control throughout functional assays.
- Testing only PMA is not sufficient to claim unaltered platelet functionality. Please include physiological platelet activators (thrombin / ADP / alcollagen-related agonists) to assess activation.
- Morphological validation is required. Fluorescence microscopy and/or electron microscopy should document platelet-like particle morphology (size distribution, granule content, and presence of a marginal microtubule ring).
- The claim of "unaltered platelet functionality" (Line 219-220) requires additional functional readouts beyond marker expression/limited activation tests (adhesion under flow, aggregation, spreading, and/or clot retraction benchmarked directly against isolated platelets and untreated control).

5. Characterization of JI051-induced proplatelets needs to be more rigorous (Figure 4).

- Based on the provided videos, JI051-induced proplatelets appear morphologically altered (finer protrusions, smaller bulges). Please add cytoskeletal characterization (actin and microtubules) to support and interpret these observations.
- Provide more details on quantification of proplatelet formation. Please state how many images and/or cells were analyzed per condition and per replicate, and describe the sampling strategy (random fields?).
- The Discussion suggests that "JI051 induced PPF not only in enlarged cells but also in a subset of cells with relatively smaller sizes." Please provide direct evidence (quantification stratified by cell size, with representative images).

Minor comments
- Please ensure all figure legends define n, specify whether replicates are biological or technical, and state statistical tests used.
- Consider "platelet-like particles" vs "platelets" to avoid overstating equivalence unless fully supported by benchmarking
- The Methods section lacks essential information for qPCR (Fig. 2E and Fig. S3). Please provide sample preparation, RNA input amounts, reverse transcription conditions, primer sequences.
- How are platelet-like particle numbers quantified (gating strategy, counting beads, instrument settings).
- Fig. S3B: Please clarify whether the shaking protocol was also applied to the knockdown experiments. If not, please provide justification and discuss comparability.

Reviewer #2 (Comments to the Authors (Required)):

This is a good study of the effects of a small molecule, JI051, on megakaryocyte maturation and platelet generation from human imMKCL cells. The setting for the manuscript is an interesting analysis of maturation-dependent changes in gene (protein) expression, in these cells, and how these changes parallel changes in cell size. 7 genes are shown to overlap in terms of expression change as the cells mature, and as they grow larger. This latter analysis is revealing, potentially, of novel drivers of MK maturation, and one of these genes, PHB2, shows a drop in expression which may drive proplatelet extension and platelet formation. The inhibitor JI051 interacts with and blocks activity of PHB2, and has been shown in a previous publication to arrest cells in G2 phase of cell cycle. The manuscript is well written and the experiments well described.

Comments to address
1. Can the authors provide more detail about the chemical nature of JI051, and its binding potential to PHB2? How does it bind and would it inhibit its activity?
2. I was not sure that the controls for the PHB2 knockout by Crispr were included in the study. Please clarify the controls.
3. Are the platelets generated in the study, by turbulence, fully characterised as platelets, rather than as platelet-like particles? In particular, can the authors show that they are negative for nuclear material, and are viable and intact, using something like a calcein dye?
4. Can the authors show specificity of the molecule JI051 by showing that it no longer has an effect on platelet generation when used in the PHB2 knockout iMMKCL line?

Reviewer #3 (Comments to the Authors (Required)):

Producing platelets in vitro remains challenging. In this study, the authors perform a proteomic analysis of iPSC-derived immortalized megakaryocyte cell lines (imMKCLs) to identify pathways that can be targeted to enhance proplatelet formation and, consequently, platelet production. Overall, the work is well structured and clearly presented; however, a few minor concerns/suggestions could further strengthen the manuscript:

1. The authors should provide clearer evidence of successful sorting/separation. The manuscript reports FACS sorting of imMKCLs at Day 3 (OFF phase) into small/medium/large fractions based on FSC (top 10%, middle 10%, bottom 10%), and the same approach at Day 6 to obtain six populations. It would be helpful to include representative pre-sort vs post-sort (re-analysis) plots (e.g., FSC histograms/overlays or dot plots) and, if available, post-sort purity metrics, to give direct visual confirmation that the three fractions are well separated at both time points.

2. Since PHB2 is a central node in the proposed biology, adding immunofluorescence staining to show how PHB2 expression/localization changes across Day 3 vs Day 6 and across small/medium/large cells would provide stronger support for the interpretation and improve the mechanistic readout.

3. Improved proplatelet formation and platelet production by imMKCLs can be achieved under turbulent conditions. However, it is not clear which results were obtained under static conditions and which under shaking. The authors should provide evidence on whether turbulence and JI051 have additive effects and whether turbulence affects PHB2. For instance, it would be of great interest to determine whether JI051 exerts an additive (or synergistic) effect under turbulent conditions in terms of proplatelet and platelet yields. In addition, assessing whether turbulence changes PHB2 expression (qPCR/flow/western blot) could help clarify whether turbulence acts by stimulating this pathway.

RE: Life Science Alliance manuscript #LSA-2026-03636

Dr. Tim Fessenden
Executive Editor
Life Science Alliance

June 12, 2026

Dear Dr. Fessenden,

Please find herewith our revised manuscript entitled "**Proteomics of heterogeneous megakaryocytes identifies JI051 as an enhancer of platelet biogenesis**" by Taga R. et al., which we submit to be considered for publication as a Research Article in ***Life Science Alliance***.

In this letter, we respond to the valuable comments from three reviewers. We sincerely thank the reviewers for their careful evaluation and constructive comments, which have substantially improved the clarity, rigor, and balance of the manuscript.

In summary, the revised manuscript clarifies the sorting strategy and improves the reproducibility of the size-fractionation analysis, tempers interpretations that are not directly supported by the present data, strengthens the figure legends and Methods section, refines the description of platelet-related phenotypes to avoid overstatement, and adds clarifications and supplementary evidence in response to the reviewers' concerns. We have also revised the Discussion section to distinguish more clearly between experimentally supported findings and speculative mechanisms and to clarify the interpretation of PHB2- and JI051-related effects on megakaryocyte maturation and platelet-like particle production.

Below, we provide a point-by-point response to each comment and summarize the corresponding revisions made in the manuscript.

Reviewer #1 (Remarks to the Author):

In their manuscript, Taga et al. address an important bottleneck for ex vivo platelet manufacturing from iPSC-derived immortalized megakaryocyte cell lines (imMKCLs), namely cellular heterogeneity that limits efficient maturation and platelet release. By combining size-stratified proteomics with principal component analysis, the authors identify prohibitin-2 (PHB2) as a putative inhibitory node and test JI051 (reported to interact with PHB2) as a simple culture additive. The authors report that JI051 added during maturation accelerates proplatelet formation and increases platelet-like particle yield (approximately three-fold under optimal timing/dose). Overall, identifying JI051 as a potential modulator that increases platelet output in imMKCLs is interesting and could have clinical relevance. However, the current manuscript lacks critical methodological detail and a sufficiently rigorous phenotypic/functional characterization to support the strength of the claims. While a deep mechanistic dissection may be beyond scope, a more comprehensive characterization of the observed phenotype is required prior to publication.

(Response)

We appreciate Reviewer #1's careful reading of our manuscript and the constructive suggestions. In response, we have clarified key methodological details, moderated interpretations that were not directly supported by the present data, strengthened the figure legends and Methods section, provided additional clarification regarding the phenotypic characterization of platelet-like particles (PLPs), and revised the text throughout to more accurately reflect the level of evidence provided in this study.

Major comments

1. Definition and validation of "cell size fractions" line 102): Please report the mean actual cell size (diameter) for each fraction and include representative images of the sorted cells. It is unclear whether the gating strategy was kept identical between the two timepoints or adjusted to capture the top/bottom 10% at each timepoint independently. Please clarify explicitly and expand the Methods to describe the gating strategy in sufficient detail for reproducibility.

(Response)

To improve clarity and reproducibility, we have added a detailed description of the gating strategy to the revised manuscript (lines 104–108), the Methods (lines 452–454), and the supplementary materials (new Fig S1A). Specifically, the top 10%, middle 10%, and bottom 10% gates were defined within the CD41^{Hi}/CD42b^{Hi} population at Day 3, and the same gating strategy was kept identical for the Day 6 samples. We have added a representative pre-sort plot and gating scheme in new Fig S1A. Because we prioritized

securing sufficient material for proteomic analysis, we did not perform post-sort flow cytometric re-analysis or imaging of the sorted cells in the original experiment. Accordingly, direct measurements of cell diameter and representative images of the sorted fractions are not available. Instead, we estimated the protein amount recovered per sorted event and found that the recovered protein increased in accordance with FSC-A intensity, supporting the conclusion that the intended relatively small, medium, and large fractions were predominantly collected, as shown in new Fig S1B and the revised manuscript (lines 110–116).

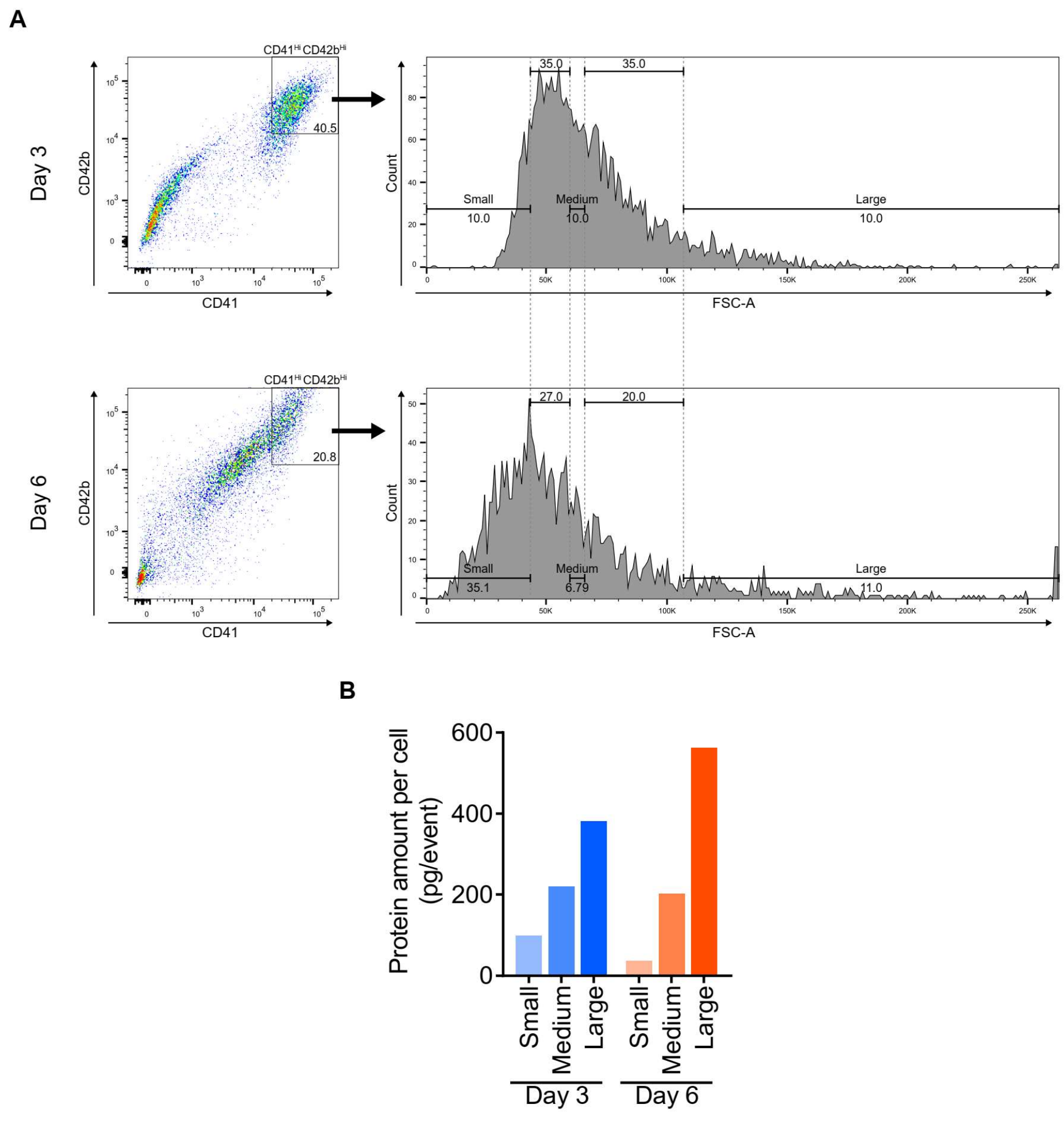

New Supplementary Figure 1

Sorting strategy of the imMKCL megakaryocytes

(A) Gating strategy. Gates corresponding to the top 10%, middle 10%, and bottom 10% of the FSC distribution were defined for the CD41^{Hi}/CD42b^{Hi} population on Day 3, and the

same gate boundaries were applied to the Day 6 samples.
(B) Protein amount per sorted event. This value was calculated from the total protein amount extracted from each sample and the number of events recorded during sorting.

2. Claims about demarcation membrane system (DMS) are not supported in this manuscript (line 146). The statement that large cells at Day 6 exhibit well-developed demarcation membrane systems indicative of readiness for platelet release is not demonstrated in the current work. If this conclusion is based on prior publications, please cite them clearly. If it is meant as an observation from this study, then representative ultrastructural evidence (or appropriate imaging) should be included.

(Response)

We agree with the reviewer that our original wording was too strong. Because we did not directly assess the ultrastructure of the demarcation membrane system in the present study, we have removed the statement that large cells at Day 6 exhibit well-developed demarcation membrane systems. Instead, we now use more cautious wording to state that the enrichment of membrane-related GO terms in the large-cell cluster may be indicative of a state compatible with readiness for platelet release (lines 158–160). Although previous work (Ito et al., *Cell*, 2018) and our preliminary observations (Fig 1 for editors) suggest that imMKCL megakaryocytes can form DMS-like structures during maturation, these findings were not evaluated as part of the present study and therefore do not provide direct support for the original statement. We therefore deleted the DMS-related interpretation from the manuscript and revised the relevant passages accordingly.

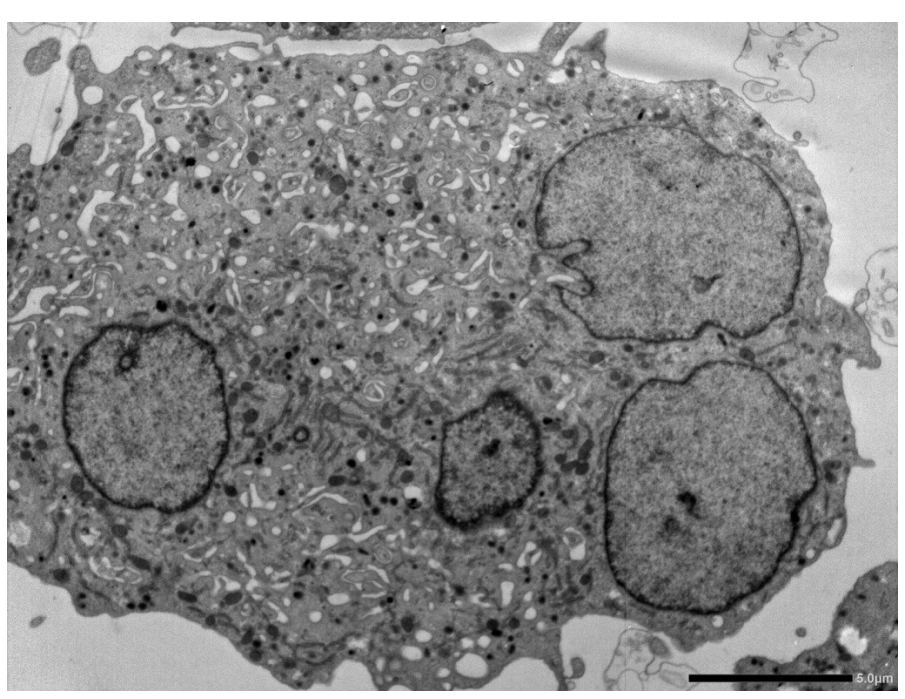

Figure 1 for editors
A representative electron microscopy image of an imMKCL cell on Day 3 of the DOX-OFF stage. Scale bar, 5µm.

3. Incomplete reporting and statistics in Fig. 3. The JI051 concentration used in the time-series

experiment is not specified (please report in the figure legend). Statistical testing is missing for Fig. 3A. Please include appropriate tests. Dose-response characterization is incomplete (Fig. 3C).

The effect of concentrations >10 µM is not shown. Does the platelet-like particle yield plateau, continue to increase, or decline (toxicity/off-target effects)?

(Response)

We appreciate this helpful comment and have revised Fig 3 and its legend accordingly. We now specify in the figure legend that the JI051 concentration used in the time-series experiment in Fig 3A was 10 µM (line 733). We also added statistical analysis for Fig 3A using Dunnett's multiple comparison test versus the JI051-untreated control based on a one-way ANOVA model, and now indicate in the new Fig 3A that the addition of JI051 on Days 2, 3, and 4 resulted in significant effects. In addition, we performed a preliminary evaluation at higher JI051 concentrations (30 and 100 µM). PLP yield did not increase further above 10 µM and instead tended to decrease at higher concentrations (Fig 2 for editors), with visible compound precipitation observed beginning at 30 µM. These observations suggest that the maximal effect is reached around 10 µM under the tested conditions, whereas higher concentrations may reduce PLP yield. Because precipitation was observed at higher concentrations, we cannot determine from the present data whether the reduction in yield reflects limited solubility, concentration-dependent toxicity, off-target effects, or a combination of these factors.

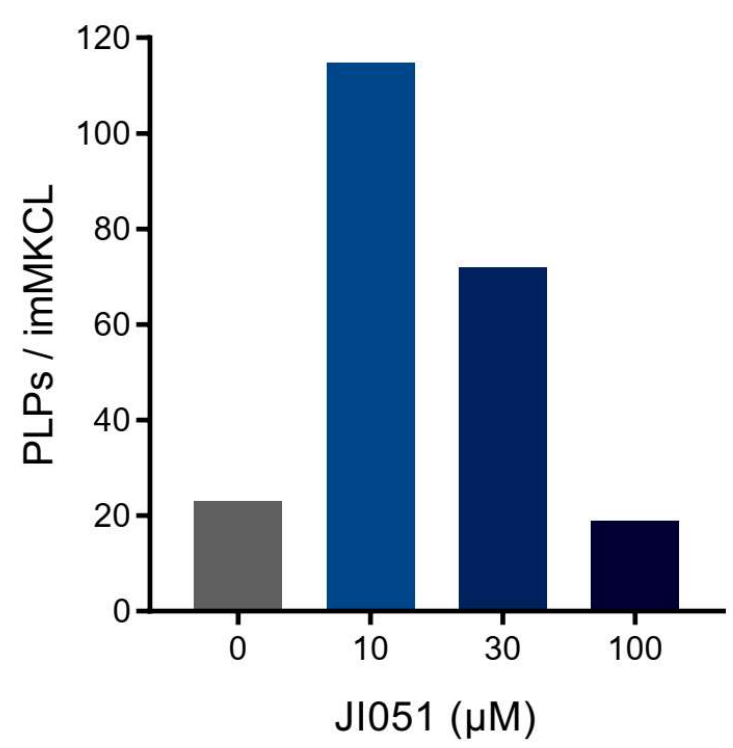

Figure 2 for editors

Number of CD41$^+$, CD42b$^+$ PLPs generated from the imMKCL cells in a shaker flask culture at the higher concentrations of JI051.

4. Platelet-like particle benchmarking and functional assessment are currently insufficient (Fig. 3D-G).

- Please include freshly isolated platelets as an additional benchmark control throughout functional assays.

- Testing only PMA is not sufficient to claim unaltered platelet functionality. Please include physiological platelet activators (thrombin / ADP / alcollagen-related agonists) to assess activation.

- Morphological validation is required. Fluorescence microscopy and/or electron microscopy should document platelet-like particle morphology (size distribution, granule content, and presence of a marginal microtubule ring).

- The claim of "unaltered platelet functionality" (Line 219-220) requires additional functional readouts beyond marker expression/limited activation tests (adhesion under flow, aggregation, spreading, and/or clot retraction benchmarked directly against isolated platelets and untreated control).

(Response)

We agree that the benchmarking and functional assessment in the original manuscript were insufficient to support a strong claim of equivalence to native platelets. Accordingly, throughout the revised manuscript, we have replaced the term "platelets" with "platelet-like particles (PLPs)" where appropriate, as also suggested in the minor comments. In addition, we have removed the original statement that JI051 does not impair platelet functionality and instead adopted more cautious wording, indicating only that PLPs generated in the presence of JI051 exhibit basic activation responses under the limited conditions examined in this study (lines 34–35 and 233–234).

With regard to the functionality of the imMKCL-derived PLPs and their benchmarking against freshly isolated platelets, these aspects have been extensively characterized in previous studies (e.g., Ito et al., *Cell*, 2018; Nakamura et al., *PLoS One*, 2025). In these previous reports, imMKCL-derived PLPs have been shown to exhibit activation of surface markers in response to physiological agonists such as ADP/TRAP-6, aggregation responses comparable to donor-derived platelets under ADP plus collagen stimulation, and intact structures including α-granules and marginal microtubule bands, although larger in size (3–8 µm) than circulating platelets. These particles have also been demonstrated to possess in vivo hemostatic activity in mouse and rabbit models.

We additionally provide supportive preliminary data regarding the properties of PLPs generated in the presence of JI051. Specifically:

- PLPs generated with JI051 retain granule-containing ultrastructural features comparable to those generated without JI051 (Fig 3A and B for editors).

- Their size distribution is similarly observed mainly within the range of 3–8 µm, consistent with previous reports (Fig 3C for editors).

- JI051-generated PLPs show increased expression of activation-related surface markers in response to stimulation with ADP/TRAP-6 (Fig 3D for editors).

- We confirmed that JI051-generated PLPs exhibit aggregation responses upon stimulation with collagen or TRAP-6 (Fig 3E and F for editors).

Although we have performed these preliminary evaluations of the properties of the imMKCL-derived PLPs, we agree with the reviewer that comprehensive benchmarking against freshly isolated platelets and full functional characterization were not completed in the present study. Therefore, we have revised the manuscript conservatively and consistently refer to these particles as PLPs, without claiming full equivalence to native platelets.

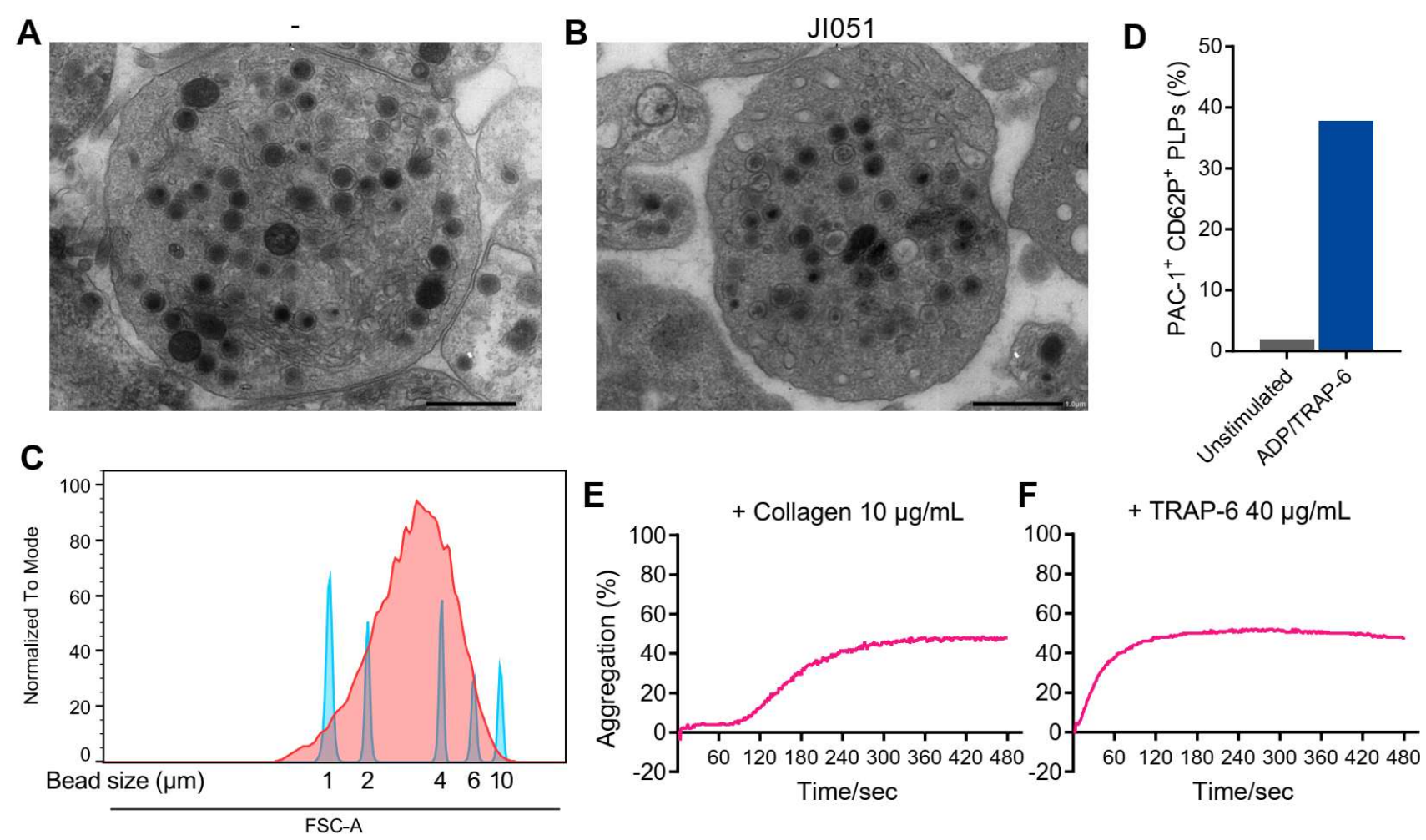

Figure 3 for editors

(A, B) Representative electron microscopy images of the imMKCL-derived PLPs (A) without JI051 or (B) with JI051. Scale bar, 1 µm. (C) Representative FSC-A plot of the PLPs produced by JI051 (red histogram). Blue histogram indicates calibration beads of known diameters (1, 2, 4, 6, and 10 µm; Thermo Fisher Scientific), which were added to the sample and measured simultaneously as size references. (D) Percentage of PAC-1^{+} CD62P^{+} PLPs generated in the presence of JI051 after stimulation with 100 µM ADP and 100 µM TRAP-6. (E, F) Aggregation of JI051-induced PLPs based on light transmission after agonist stimulation by (E) 10 µg/mL collagen or (F) 40 µg/mL TRAP-6.

5. Characterization of JI051-induced proplatelets needs to be more rigorous (Figure 4).

- Based on the provided videos, JI051-induced proplatelets appear morphologically altered

(finer protrusions, smaller bulges). Please add cytoskeletal characterization (actin and microtubules) to support and interpret these observations.

- Provide more details on quantification of proplatelet formation. Please state how many images and/or cells were analyzed per condition and per replicate, and describe the sampling strategy (random fields?).

- The Discussion suggests that "JI051 induced PPF not only in enlarged cells but also in a subset of cells with relatively smaller sizes." Please provide direct evidence (quantification stratified by cell size, with representative images).

(Response)

We agree that the structures induced by JI051 may not fully correspond to conventionally defined proplatelets. Because we did not perform cytoskeletal analyses of actin or microtubules in this study, we have replaced the term "proplatelet" with the more descriptive term "proplatelet-like extensions" throughout the revised manuscript. We also clarified the quantification procedure in the Methods (lines 496–500). Briefly, bright-field images were acquired from the central area of each well, and approximately 50 cells were evaluated per condition and per replicate for the presence or absence of proplatelet-like extensions. The evaluation was performed in a manner blinded to conditions.

Finally, we agree that our previous statement that JI051 may induce these structures in relatively smaller cells was not sufficiently supported. Because this interpretation was based solely on qualitative observations from the IncuCyte data, and quantitative analysis stratified by cell size was not performed in the present study, we omitted this statement from the Discussion (lines 322–323).

Minor comments

- Please ensure all figure legends define n, specify whether replicates are biological or technical, and state statistical tests used.

(Response)

We have revised all figure legends to define n, specify whether replicates are biological or technical, and state the statistical tests used, including in the new Fig S4. We have added a corresponding description to the Statistical Analysis section of the Methods in the revised manuscript (lines 518–529).

- Consider "platelet-like particles" vs "platelets" to avoid overstating equivalence unless fully supported by benchmarking

(Response)

We agree with this point and have revised the terminology throughout the manuscript.

Because benchmarking was not sufficient to support full equivalence to native platelets, we now use the term "platelet-like particles (PLPs)" instead of "platelets" where appropriate throughout the manuscript.

- The Methods section lacks essential information for qPCR (Fig. 2E and Fig. S3). Please provide sample preparation, RNA input amounts, reverse transcription conditions, primer sequences.

(Response)

We have added essential details for the qPCR experiments to the Methods section, including sample preparation, RNA input amounts, reverse transcription conditions, and TaqMan assay information (lines 432–443). Because commercially supplied TaqMan assays were used, primer sequences are proprietary and are therefore not available; the corresponding assay identifiers are now provided.

- How are platelet-like particle numbers quantified (gating strategy, counting beads, instrument settings).

(Response)

We have added a description of how platelet-like particle numbers were quantified in the Methods, including the gating strategy, Trucount bead-based quantification approach, flow cytometry acquisition criteria, and relevant measurement conditions (lines 409–420).

- Fig. S3B: Please clarify whether the shaking protocol was also applied to the knockdown experiments. If not, please provide justification and discuss comparability.

(Response)

The knockdown experiments shown in new Fig S4 were performed under static conditions, and we have clarified this in the revised manuscript (line 190). The reason is that the shaking protocol requires substantially more viable cells than can be reliably obtained after gRNA/Cas9-mediated manipulation. We attempted to apply the shaking protocol after an extended expansion period following transduction; however, under these conditions, the PHB2 knockout efficiency was markedly reduced (approximately 10%), preventing reliable evaluation. Although we were unable to directly compare the contribution of PHB2 under static and shaking conditions, PHB2 expression itself showed a similar trend across these culture conditions (Fig 4 for editors). Therefore, although the comparability of the functional effects cannot be assessed directly from the present data, the overall pattern of PHB2 expression does not appear to differ substantially between static and shaking culture conditions.

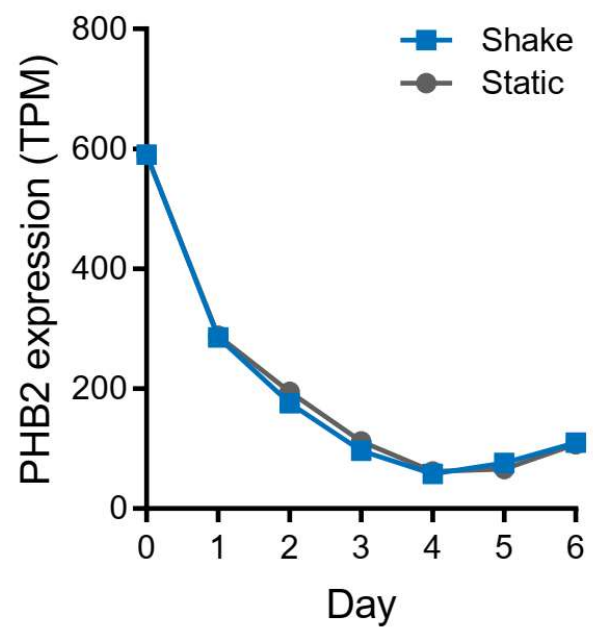

Figure 4 for editors

Changes in PHB2 mRNA expression during imMKCL maturation under DOX-OFF conditions in static and shaking cultures.

Reviewer #2 (Remarks to the Author):

This is a good study of the effects of a small molecule, JI051, on megakaryocyte maturation and platelet generation from human imMKCL cells. The setting for the manuscript is an interesting analysis of maturation-dependent changes in gene (protein) expression, in these cells, and how these changes parallel changes in cell size. 7 genes are shown to overlap in terms of expression change as the cells mature, and as they grow larger. This latter analysis is revealing, potentially, of novel drivers of MK maturation, and one of these genes, PHB2, shows a drop in expression which may drive proplatelet extension and platelet formation. The inhibitor JI051 interacts with and blocks activity of PHB2, and has been shown in a previous publication to arrest cells in G2 phase of cell cycle. The manuscript is well written and the experiments well described.

(Response)

We thank Reviewer #2 for the positive assessment of our study and for the helpful comments. We are encouraged that the reviewer found the proteomic analysis and the identification of PHB2 as a candidate regulator of megakaryocyte maturation to be of interest. In response to the comments below, we have clarified the description of JI051 and its relationship to PHB2, added details regarding the knockout controls, and revised the manuscript to describe more carefully the characterization of platelet-like particles and the scope of our conclusions.

1. Can the authors provide more detail about the chemical nature of JI051, and its binding potential to PHB2? How does it bind and would it inhibit its activity?

(Response)

We have added a description of the chemical nature of JI051 to the revised manuscript based on previous reports (lines 202–205). JI051 is a small-molecule compound with an

indole-based scaffold and has been described as a modulator of protein–protein interactions involving PHB2. Previous studies support a physical association between JI051 and PHB2; however, the precise binding mode remains unclear (Perron et al., *JBC*, 2018; Yang et al., *Nat. Commun.*, 2022).
We also attempted to predict the interaction between PHB2 and JI051 in silico; however, the confidence was low, and the binding mode could not be predicted reliably. In addition, PHB2 typically functions as part of a larger prohibitin complex (Hong et al., *PNAS*, 2025), making it difficult to infer the detailed mode of interaction between PHB2 and JI051. Thus, while PHB2 was identified in our analysis as a putative inhibitory node, our data do not support a definitive mechanistic interpretation of JI051 as a direct PHB2 inhibitor. Specifically, PHB2 partial knockout itself did not enhance PLP production (Fig S4B). In addition, as described in our response to Reviewer #2 Comment #4 below, the effect of JI051 on PLP production was not attenuated by PHB2 partial knockout, suggesting that the action of JI051 may involve additional targets or a broader pathway rather than direct inhibition of PHB2 alone.

2. I was not sure that the controls for the PHB2 knockout by Crispr were included in the study. Please clarify the controls.

(Response)

We appreciate this comment and agree that the description of the controls was insufficient in the original version. We have now clarified the experimental design in the Methods section (lines 391–396). Specifically, we state that a scrambled sgRNA mixture composed of three non-targeting sgRNAs was used as the negative control, and that the PHB2-targeting condition used an equimolar mixture of three PHB2 sgRNAs. We have also provided the sgRNA sequence information in the new Table S9.

3. Are the platelets generated in the study, by turbulence, fully characterised as platelets, rather than as platelet-like particles? In particular, can the authors show that they are negative for nuclear material, and are viable and intact, using something like a calcein dye?

(Response)

We agree that the particles generated in this study should be described conservatively unless they are fully benchmarked against native platelets. As noted in our response to Reviewer #1 Comment #4, previous studies of imMKCL-derived platelet-like particles (PLPs) have demonstrated activation responses to physiological agonists, aggregation capacity, ultrastructural features consistent with platelet identity, and in vivo hemostatic activity. In addition, our preliminary data further support the platelet-like properties of

JI051-induced PLPs, including electron microscopy images, activation responses to physiological agonists, and aggregation capacity (Fig 2 for editors).

In the present study, we additionally confirmed that PLPs generated with or without JI051 are negative for nuclear staining with DRAQ5, whereas the remaining imMKCL population is DRAQ5-positive (Fig 5A for editors). We also examined JI051-generated PLPs using calcein staining and observed that most CD41$^+$, CD42b$^+$ PLPs were calcein-positive, consistent with these particles being viable and structurally intact (Fig 5B for editors). Because comprehensive benchmarking remains incomplete, we have revised the manuscript to refer to these particles as PLPs rather than asserting full equivalence to circulating platelets.

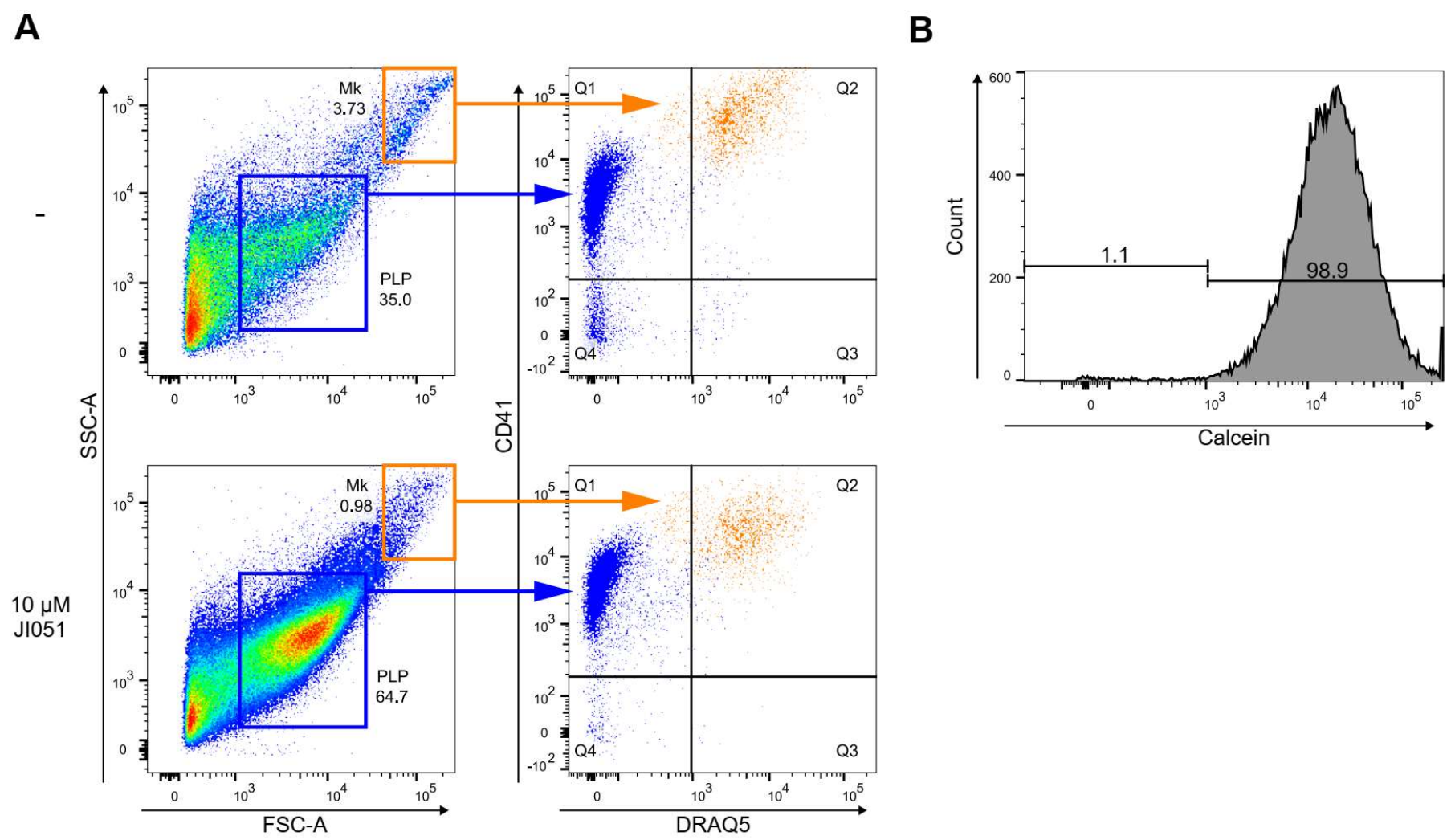

Figure 5 for editors

(A) Representative plot of PLPs (blue) and remaining imMKCL cells (orange) stained with DRAQ5. (B) Representative histogram of CD41$^+$ CD42b$^+$ PLPs stained with calcein.

4. Can the authors show specificity of the molecule JI051 by showing that it no longer has an effect on platelet generation when used in the PHB2 knockout iMMKCL line?

(Response)

We also considered this an important question and performed the suggested experiment. Specifically, we tested the effect of 10 µM JI051 in imMKCL megakaryocytes with partial PHB2 knockout. The effect of JI051 on PLP production was not diminished by PHB2 partial knockout (Fig 6 for editors). Although the knockout efficiency was approximately 40% and therefore does not exclude a contribution from residual PHB2, this finding suggests that the activity of JI051 may not be explained solely by inhibition of PHB2.

Rather, JI051 may act through one or more additional, as yet unidentified molecules or pathways that contribute to enhanced platelet production.

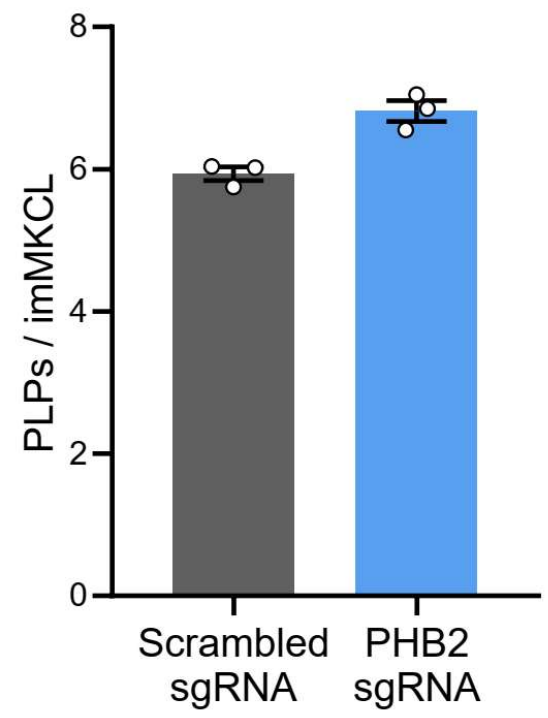

Figure 6 for editors

Number of JI051-induced CD41^{+} CD42b^{+} PLPs generated from partial knockout cells in static dish culture.

Reviewer #3 (Remarks to the Author):

Producing platelets in vitro remains challenging. In this study, the authors perform a proteomic analysis of iPSC-derived immortalized megakaryocyte cell lines (imMKCLs) to identify pathways that can be targeted to enhance proplatelet formation and, consequently, platelet production. Overall, the work is well structured and clearly presented; however, a few minor concerns/suggestions could further strengthen the manuscript:

(Response)

We thank Reviewer #3 for the thoughtful suggestions and constructive comments. In response, we have clarified several methodological and interpretive points, added supporting information where appropriate, and revised the manuscript to improve clarity and accuracy throughout.

1. The authors should provide clearer evidence of successful sorting/separation. The manuscript reports FACS sorting of imMKCLs at Day 3 (OFF phase) into small/medium/large fractions based on FSC (top 10%, middle 10%, bottom 10%), and the same approach at Day 6 to obtain six populations. It would be helpful to include representative pre-sort vs post-sort (re-analysis) plots (e.g., FSC histograms/overlays or dot plots) and, if available, post-sort purity metrics, to give direct visual confirmation that the three fractions are well separated at both time points.

(Response)

We agree that direct visual confirmation of successful separation would be helpful. However, in the original proteomics experiment, we prioritized securing enough sorted

cells for downstream protein extraction and therefore did not perform post-sort flow cytometric re-analysis or imaging of the sorted fractions. Instead, we assessed the amount of recovered protein per sorted event and found that the recovered protein increased in accordance with FSC, supporting the conclusion that the collected cell fractions indeed reflected the targeted cell sizes (new Fig S1B). In addition, we have now added a representative pre-sort plot from an independent experiment to new Fig S1A, together with the gating strategy, to clarify how the fractions were defined.

2. Since PHB2 is a central node in the proposed biology, adding immunofluorescence staining to show how PHB2 expression/localization changes across Day 3 vs Day 6 and across small/medium/large cells would provide stronger support for the interpretation and improve the mechanistic readout.

(Response)

We agree that immunofluorescence-based evaluation of PHB2 expression and localization would provide useful mechanistic information. We attempted immunostaining of matured imMKCL megakaryocytes to assess PHB2 expression and localization. Although we observed a tendency for decreased staining intensity during maturation, broadly consistent with the results shown in Fig 2E and F, we were unable to obtain sufficiently robust and reproducible signals for rigorous interpretation of localization patterns or for reliable comparison among the size-based cell fractions. We therefore concluded that the current data are not adequate to support a mechanistic discussion based on PHB2 immunofluorescence. Accordingly, we have not included these analyses in the revised manuscript and instead note that further investigation may help to clarify how PHB2 contributes to platelet production during maturation (lines 338–341).

3. Improved proplatelet formation and platelet production by imMKCLs can be achieved under turbulent conditions. However, it is not clear which results were obtained under static conditions and which under shaking. The authors should provide evidence on whether turbulence and JI051 have additive effects and whether turbulence affects PHB2. For instance, it would be of great interest to determine whether JI051 exerts an additive (or synergistic) effect under turbulent conditions in terms of proplatelet and platelet yields. In addition, assessing whether turbulence changes PHB2 expression (qPCR/flow/western blot) could help clarify whether turbulence acts by stimulating this pathway.

(Response)

We have clarified in the revised figure legends that Fig 4, S4, S5, and S6 were obtained under static conditions. To examine whether the effect of JI051 on PLP yield differed

between static and shaking conditions, we conducted a post hoc analysis using the existing datasets shown in Fig 3C and Fig S5A, which were not prospectively designed to test this interaction. A two-way ANOVA model was applied with culture condition (static vs. shaking) and JI051 concentration (0, 0.1, 1, and 10 µM) as factors. A statistically significant interaction between culture condition and JI051 concentration was observed ($F_{(3, 24)} = 4.45$, $P = 0.0127$). This result indicates that the magnitude of the JI051-associated increase in PLP yield differed between culture conditions, with a more pronounced effect observed under shaking conditions. Thus, our data support the effect of JI051 on PLP yield is greater under shaking than under static culture conditions. Because proplatelet-like extensions are expected to be continuously fragmented into PLPs under turbulent conditions, direct quantitative evaluation of these structures is technically difficult in shaking cultures. Nevertheless, the increase in PLP yield is consistent with the possibility that proplatelet-like extension formation and/or conversion to PLPs is enhanced under these conditions. However, because the present experiments were not specifically designed to distinguish mechanistic synergy from condition-dependent enhancement, we have avoided making a definitive claim of biological synergy in the revised manuscript.

We also compared PHB2 expression during imMKCL maturation under static and shaking conditions. The decrease in PHB2 expression during maturation was observed similarly under both conditions, suggesting that turbulence is unlikely to substantially affect the PHB2-related pathway (Fig 4 for editors). The molecular interplay between turbulence and the JI051- and PHB2-related pathways therefore remains to be clarified in future studies.

July 27, 2026

RE: Life Science Alliance Manuscript #LSA-2026-03636R

Dr. Hideki Hayashi
Otsuka
Department of Open Innovation, Tokushima Research Center for Drug Discovery, Pharmaceutical Division
463-10 Kagasuno, Kawauchi-cho
Tokushima, Tokushima 771-0192
Japan

Dear Dr. Hayashi,

Thank you for submitting your revised manuscript entitled "Proteomics of heterogeneous megakaryocytes identifies JI051 as an enhancer of platelet biogenesis". We returned this to Reviewers 1 and 2, whose comments are included at the end of this letter. We concur with Reviewer 1 that critical observations included in Figure 3 for the editors must be included in the manuscript (if you wish, as a supplemental figure). These data may be included as shown, without replicates, in which case the text/caption should make this clear. Conversely you may add replicates if you wish. In accordance with the reviewer support, we would be happy to publish your paper in Life Science Alliance pending this addition and final revisions necessary to meet our formatting guidelines.

MANUSCRIPT ORGANIZATION AND FORMATTING:

To avoid unnecessary delays in the acceptance and publication of your paper, please read the following information carefully. Full guidelines are available on our Instructions for Authors page, https://www.life-science-alliance.org/authors

Submission of a paper that does not conform to Life Science Alliance guidelines will delay the acceptance of your manuscript.

-Please add ORCID ID for corresponding author - you should have received instructions on how to do so.
-Please add the X and Bluesky handles of your host institute/organization, as well as your own, and/or one of the authors, in our system.
-Please add callouts for Figures S2A-D; S3A-B; S4A-B and S5A-B to your main manuscript text.
-Please upload RNA seq and proteomics data to the appropriate repositories and include accession numbers for these data in the Data Availability section.
-LSA does not permit a Supplemental Methods section. Please add the methods here to the main methods section.

We encourage our authors to provide original source data, particularly uncropped/-processed electrophoretic blots and spreadsheets for the main figures of the manuscript. If you would like to add source data, we would welcome one PDF/Excel-file per figure for this information. These files will be linked as supplementary "Source Data" files.

It is Life Science Alliance policy that if requested, original data images must be made available to the editors. Failure to provide original images upon request will result in unavoidable delays in publication. Please ensure that you have access to all original data images prior to final submission.

If you are planning a press release on your work, please inform us immediately to allow informing our production team and scheduling a release date.

We welcome submissions of potential cover images for the issue of LSA in which your work would appear. If you have high quality images associated with this work, please feel free to email these, with a caption, to the journal office.

LSA encourages authors to provide a 30-60 second video where the study is briefly explained. These videos will be appear embedded with the manuscript online at Life Science Alliance, and on social media to promote the published paper and authors (for examples, see https://docs.google.com/document/d/1-UWCfbE4pGcDdcgzcmiuJl2XMBJnxKYeqRvLLrLSo8s/edit?usp=sharing). Corresponding or first-authors are welcome to submit the video. Please submit only one video per manuscript. The video can be emailed to contact@life-science-alliance.org

FINAL FILES:

To upload the final version of your manuscript, please log in to your account: https://lsa.msubmit.net/cgi-bin/main.plex
You will be guided to complete the submission of your revised manuscript and to fill in all necessary information. Please get in

touch in case you do not know or remember your login name.

The following items are required for acceptance.

-- An editable version of the final text (.DOC or .DOCX) is needed for copyediting (no PDFs).

-- High-resolution figure, supplementary figure and video files uploaded as individual files: See our detailed guidelines for preparing your production-ready images, https://www.life-science-alliance.org/authors

-- Summary blurb (enter in submission system): A short text summarizing in a single sentence the study (max. 200 characters including spaces). This text is used in conjunction with the titles of papers, hence should be informative and complementary to the title. It should describe the context and significance of the findings for a general readership; it should be written in the present tense and refer to the work in the third person. Author names should not be mentioned.

The license to publish form must be signed before your manuscript can be sent to production. A link to the license to publish form will be available to the corresponding author only. Please take a moment to check your funder requirements.

Reviews, decision letters, and point-by-point responses associated with peer-review at Life Science Alliance will be published, alongside the manuscript. If you do want to opt out of having the reviewer reports and your point-by-point responses displayed, please let us know immediately.

Thank you for your attention to these final processing requirements. Please revise and format the manuscript and upload materials as soon as you are able.

Thank you for this interesting contribution to the literature. We look forward to publishing your paper in Life Science Alliance.

Sincerely,

Tim Fessenden
Scientific Editor
Life Science Alliance

Reviewer #1 (Comments to the Authors (Required)):

The authors have addressed all of my questions in the revised manuscript, and I recommend the manuscript for publication in Life Science Alliance. Given the important additional data presented in "Figure 3 for Editors," I suggest adding replicates to enable statistical analysis and incorporating these data into the manuscript.

Reviewer #2 (Comments to the Authors (Required)):

This study, now in its revised form, describes the effects of a small molecule, JI051, on megakaryocyte maturation and platelet generation from human imMKCL cells. The setting for the manuscript is an interesting analysis of maturation-dependent changes in gene (protein) expression, in these cells, and how these changes parallel changes in cell size. 7 genes are shown to overlap in terms of expression change as the cells mature, and as they grow larger. This latter analysis is revealing, potentially, of novel drivers of MK maturation, and one of these genes, PHB2, shows a drop in expression which may drive proplatelet extension and platelet formation. The inhibitor JI051 interacts with and blocks activity of PHB2, and has been shown in a previous publication to arrest cells in G2 phase of cell cycle. The manuscript is well written and the revisions to the manuscript improve it.
The authors have addressed the comments made in the original submission. In particular they have provided a description of the chemistry of JI051, although it was interesting to see that knockdown of PHB2 did not affect the response to JI051, suggesting that there may be other targets. This is discussed in the manuscript. Controls for Crispr are now described in the manuscript. The platelet-like particles generated are also now described as such. In all, these are satisfactory revisions to the manuscript.

Re: Editorial Decision LSA-2026-03636R

Dr. Tim Fessenden
Executive Editor
Life Science Alliance

September 08, 2026

Dear Dr. Fessenden,

Thank you for your positive evaluation of our manuscript entitled "**Proteomics of heterogeneous megakaryocytes identifies JI051 as an enhancer of platelet biogenesis.**" We are pleased to submit the final revised version of manuscript LSA-2026-03636R.

Following your recommendation and Reviewer 1's suggestion, we have added the data previously presented as "Figure 3 for Editors" to the manuscript as Figure S6. The main text and figure legend now state that these data were obtained from a single liter-scale culture experiment (n = 1).

We have also completed the other revisions requested in the decision letter, including:

- addition of the missing Supplementary Figure callouts to the main text,
- deposition of the proteomics and RNA-seq datasets and inclusion of accession numbers in the Data Availability section,
- transfer of methods previously provided in the Supplemental Methods to the main Materials and Methods section, and
- final formatting revisions in accordance with Life Science Alliance guidelines.

We also identified a typographical error in the previously submitted Response to Reviewer document. In the description of "Figure 3 for Editors," the concentration in panel (F) was incorrectly given as "40 μg/mL TRAP-6" instead of "40 μM TRAP-6." We have corrected this error and have attached the corrected figure below as the "New Figure 3 for Editors." The newly added Figure S6 also shows the correct unit.

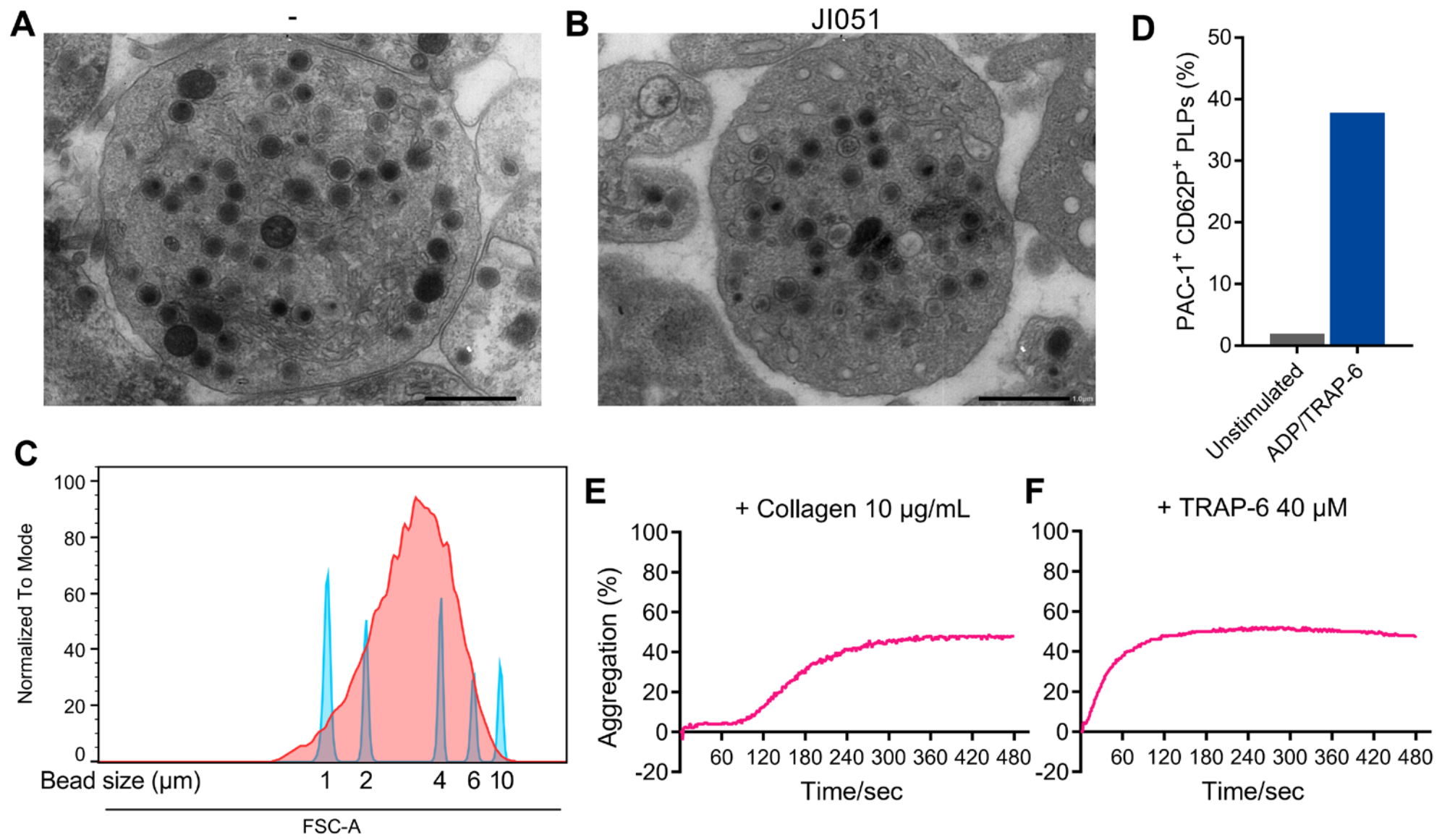

New Figure 3 for Editors

(A, B) Representative electron microscopy images of the imMKCL-derived PLPs (A) without JI051 or (B) with JI051. Scale bar, 1 µm. (C) Representative FSC-A plot of the PLPs produced by JI051 (red histogram). Blue histogram indicates calibration beads of known diameters (1, 2, 4, 6, and 10 µm; Thermo Fisher Scientific), which were added to the sample and measured simultaneously as size references. (D) Percentage of PAC-1^{+} CD62P^{+} PLPs generated in the presence of JI051 after stimulation with 100 µM ADP and 100 µM TRAP-6. (E, F) Aggregation of JI051-induced PLPs based on light transmission after agonist stimulation by (E) 10 µg/mL collagen or (F) 40 µM TRAP-6.

September 8, 2026

RE: Life Science Alliance Manuscript #LSA-2026-03636RR

Dr. Hideki Hayashi
Otsuka
Department of Open Innovation, Tokushima Research Center for Drug Discovery, Pharmaceutical Division
463-10 Kagasuno, Kawauchi-cho
Tokushima, Tokushima 771-0192
Japan

Dear Dr. Hayashi,

Thank you for submitting your Research Article entitled "Proteomics of heterogeneous megakaryocytes identifies JI051 as an enhancer of platelet biogenesis". It is a pleasure to let you know that your manuscript is now accepted for publication in Life Science Alliance. Congratulations on this interesting work.

The final published version of your manuscript will be deposited by us to PubMed Central upon publication.

Your manuscript will now progress through copyediting and proofing. It is journal policy that authors provide original data upon request.

Your article will publish open access upon publication under a CC-BY license.

Reviews, decision letters, and point-by-point responses associated with peer-review at Life Science Alliance will be published, alongside the manuscript. If you do want to opt out of having the reviewer reports and your point-by-point responses displayed, please let us know immediately.

IMPORTANT: If you will be unreachable at any time, please provide us with the email address of an alternate author. Failure to respond to routine queries may lead to unavoidable delays in publication.

Scheduling details will be available from our production department. You will receive proofs shortly before the publication date. Only essential corrections can be made at the proof stage so if there are any minor final changes you wish to make to the manuscript, please let the journal office know now.

DISTRIBUTION OF MATERIALS:
Authors are required to distribute freely any materials used in experiments published in Life Science Alliance. Authors are encouraged to deposit materials used in their studies to the appropriate repositories for distribution to researchers.

You can contact the journal office with any questions, contact@life-science-alliance.org

Again, congratulations on a very nice paper. I hope you found the review process to be constructive and are pleased with how the manuscript was handled editorially. We look forward to future exciting submissions from your lab.

Sincerely,

Tim Fessenden
Scientific Editor
Life Science Alliance
